# Ryanodine receptor-mediated arrhythmias and sudden cardiac death

**DOI:** 10.1016/j.pharmthera.2009.03.006

**Published:** 2009-08

**Authors:** Lynda M. Blayney, F. Anthony Lai

**Affiliations:** Wales Heart Research Institute, Cardiff University School of Medicine, Cardiff CF14 4XN, UK

**Keywords:** Ryanodine receptor, Cardiac arrhythmia, Sudden cardiac death, Heart failure, Catecholaminergic polymorphic ventricular tachycardia, AP, action potential, ARVD2, arrhythmogenic right ventricular cardiomyopathy type 2, CaMKII, Ca^2+^/calmodulim-dependent protein kinase II, CICR, Ca^2+^ induced Ca^2+^ release, CM, calmodulin, CPVT, catecholaminergic polymorphic ventricular tachycardia, CSQ, calsequestrin, DAD, delayed after depolarisation, ECC, excitation–contraction coupling, FKBP12/12.6, FK506 binding protein, HF, heart failure, LCC, L-type Ca^2+^ channel, mAKAP, muscle specific A kinase anchoring protein, NCX, Na^+^/Ca^2+^ exchange protein, P-1 or P-2, phosphatase inhibitor type-1 or type-2, PDE4D3, phosphodiesterase 4D3, PKA, protein kinase A, PLB, phosphoplamban, PP1, protein phosphatase 1, PP2A, protein phosphatase 2A, RyR1/2, ryanodine receptor type-1/type-2, SCD, sudden cardiac death, SERCA, sarcoplasmic reticulum Ca^2+^ ATPase, SL, sarcolemma, SR, sarcoplasmic reticulum, SOICR, store-overload-induced Ca^2+^ release

## Abstract

The cardiac ryanodine receptor-Ca^2+^ release channel (RyR2) is an essential sarcoplasmic reticulum (SR) transmembrane protein that plays a central role in excitation–contraction coupling (ECC) in cardiomyocytes. Aberrant spontaneous, diastolic Ca^2+^ leak from the SR due to dysfunctional RyR2 contributes to the formation of delayed after-depolarisations, which are thought to underlie the fatal arrhythmia that occurs in both heart failure (HF) and in catecholaminergic polymorphic ventricular tachycardia (CPVT). CPVT is an inherited disorder associated with mutations in either the RyR2 or a SR luminal protein, calsequestrin. RyR2 shows normal function at rest in CPVT but the RyR2 dysfunction is unmasked by physical exercise or emotional stress, suggesting abnormal RyR2 activation as an underlying mechanism. Several potential mechanisms have been advanced to explain the dysfunctional RyR2 observed in HF and CPVT, including enhanced RyR2 phosphorylation status, altered RyR2 regulation at luminal/cytoplasmic sites and perturbed RyR2 intra/inter-molecular interactions. This review considers RyR2 dysfunction in the context of the structural and functional modulation of the channel, and potential therapeutic strategies to stabilise RyR2 function in cardiac pathology.

## 1 Introduction

Ryanodine receptors (RyR) were first observed in the 1970s, as the so-called ‘foot’ structures in electron micrographs of striated muscle, spanning the gap that is found at specific junctions between the plasma membrane, or sarcolemma (SL), and the intracellular sarcoplasmic reticulum (SR) membrane (Franzini-Armstrong, 1970; Campbell et al., 1980). The RyR was subsequently isolated as an integral SR membrane protein and its role as the Ca^2+^ release channel demonstrated (Lai et al., 1988). The complementary DNA encoding three distinct RyR channels was cloned and the corresponding gene sequences obtained for three isoforms; RyR1 (Takeshima et al., 1989; Zorzato et al., 1990), RyR2 (Nakai et al., 1990; Otsu et al., 1990; Tunwell et al., 1996) and RyR3 (Hakamata et al., 1992). Through the 1980s and 1990s the central role of RyRs and the physiological characteristics of excitation–contraction coupling (ECC) have been established (Stern et al., 1999; Bers, 2002). Within the current decade, discovery of numerous RyR2 gene mutations which underlie the arrhythmogenesis that leads to sudden cardiac death (SCD) in catecholaminergic polymorphic ventricular tachycardia (CPVT), have added a new focus to the role of RyR2 dysfunction in cardiac disease (Priori et al., 2001; George et al., 2007).

RyRs are expressed in many tissues but their most clearly defined pivotal role is in the regulation of ECC in muscle cells. In the heart, ECC is the process by which an action potential (AP) at the surface of a cardiomyocyte regulates the release of Ca^2+^ ions from SR Ca^2+^ stores which then diffuse and bind to the myofilaments to initiate contraction (systole). The reuptake of Ca^2+^ into the SR Ca^2+^ store causes relaxation (diastole), to complete the cycle comprising one heart beat. The heart beat therefore is critically dependent on the fidelity of ECC, which in turn is exquisitely regulated by the signal transmission occurring through the microdomain architecture at the regions where the sarcolemma (SL) and its T-tubule invaginations directly abut the junctional SR domains (Franzini-Armstrong et al., 1999). These membranes are traversed by the critical membrane pumps, ion exchangers and ion channels, which act synergistically to maintain Ca^2+^ homeostasis and the AP. Control of cytoplasmic free Ca^2+^ ion concentration is the key to the ECC process and this vital parameter is regulated by a plethora of biochemical and protein modulators and phosphorylation (Bers, 2002, 2004; Meissner, 2004).

Both physically and functionally, the RyR2 Ca^2+^ channels preside at the heart of ECC acting as sentinels to the large SR Ca^2+^ store. This review centres upon the role of the cardiac RyR2 isoform in arrhythmia and SCD. RyR2 dysfunction manifested as tachyarrhythmias is triggered by physical or emotional stress in CPVT patients and similar symptoms causes SCD in ∼50% of heart failure (HF) patients. These arrhythmias are believed to arise as a consequence of delayed after-depolarisations (DADs) that are initiated by diastolic leak of Ca^2+^ from dysfunctional RyR2. RyR2 function in the context of its normal structural and functional regulation and its role in ECC will be discussed in this review. The mechanism(s) by which RyR2 becomes dysfunctional, including abnormal phosphorylation, disrupted interaction with regulatory proteins and ions, or altered RyR2 domain interactions will be examined, and potential strategies for therapeutic intervention will be considered.

## 2 Ryanodine receptor type-2 and excitation–contraction coupling

### 2.1 Junctional microdomain structure

**Fig. 1:**
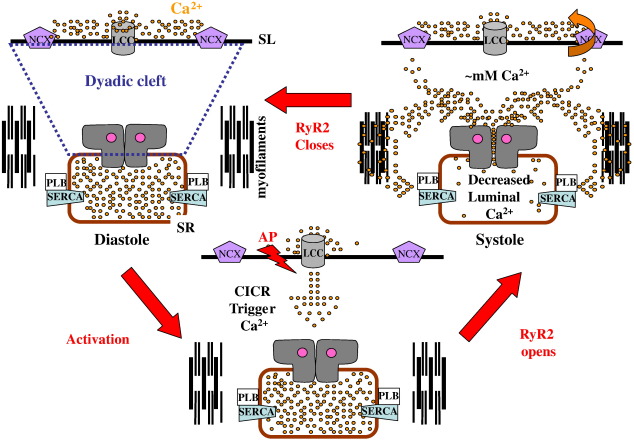
Movement of Ca^2+^ between the SR Ca^2+^ store, the dyadic space and the extracellular space during ECC. During diastole the Ca^2+^ concentration in the dyadic cleft is < 10^−7^ M, intracellular Ca^2+^ is contained within the SR, and the RyR2 channel (in grey) is closed. As the AP sweeps across the SL (‘Activation’), the voltage-sensitive LCC is activated to allow the entry of trigger Ca^2+^ (∼ 10% of that required for contraction enters the dyadic space). The rapid rise in local Ca^2+^ concentration activates RyR2 (‘RyR2 Opens’). The Ca^2+^ concentration within the dyadic space rises further as Ca^2+^ is released from the SR Ca^2+^ store, which activates the NCX and inhibits LCC. The released Ca^2+^ diffuses into the cytoplasm to deinhibit the contractile proteins and myofilament contraction occurs. Ca^2+^ is pumped back into the SR by SERCA and out of the cell via NCX, thus Ca^2+^ homeostasis is restored. RyR2 closes during systole and this could be mediated by either reduced Ca^2+^ concentrations in the SR lumen, inhibitory action of dyadic cleft Ca^2+^ concentrations or by inherent RyR2 mechanisms making it unavailable for stimulation by Ca^2+^. These events are further discussed in the text.

Of vital importance to the reliability of ECC is the myocyte intracellular architecture (Koh et al., 2006). The precise juxtaposition of the SL and SR forms specific junctional microdomains, creating a 10–12 nM gap, known as the dyadic cleft, where the RyR2 cytoplasmic region resides, and its transmembrane region spans the SR membrane to immerse the ‘luminal’ portion into the SR Ca^2+^ store (Franzini-Armstrong et al., 1999). Traversing the SL is the L-type Ca^2+^ channel (LCC) and the Na^+^/Ca^2+^ exchange protein (NCX). Although in cardiac muscle there is no allosteric contact between these proteins they are co-ordinately regulated via the changing concentrations of Ca^2+^, Na^+^ and K^+^ within the dyadic cleft (Stern, 1992; Niggli & Lipp, 1993; Greenstein & Winslow, 2002) (Fig. 1). Regular 2D arrays of RyR2 are found at the junctional SR with clusters comprising 70–250 RyR2 particles (Chen-Izu et al., 2006; Soeller et al., 2007) depending upon species (Franzini-Armstrong et al., 1999), with faster heart rate corresponding to greater numbers within an array. The RyR together with the LCC (and NCX and other ion channels) form a unit referred to as a ‘couplon’ (Stern et al., 1999). Although the term couplon describes the physical unit of a junctional domain, the functional Ca^2+^ release unit may only be a fraction of the RyR array and be defined by physiological and experimental conditions (see Section 2.2.1 on Ca^2+^ sparks).

### 2.2 Regulation of ryanodine receptor type-2 by Ca^2+^

Throughout the ECC cycle, the RyR2 is exposed to continually changing Ca^2+^ concentrations, both dyadic, luminal and within the transmembrane pore. These changes are critical to RyR2 regulation and numerous studies have modelled the precise modal gating of the RyR by Ca^2+^. These aspects are beyond the scope of this review, but have been given comprehensive treatment by others (Rice et al., 1999; Stern et al., 1999; Soeller & Cannell, 2004; Koh et al., 2006). To measure the dynamics of gating, single RyR channels are inserted into planar lipid bilayers separating two chambers; the *cis* chamber — corresponding to the cytoplasmic face of RyR, and *trans* chamber corresponding to the SR luminal face. Briefly, this method involves measuring the current flowing between the two chambers, carried by ion fluxes occurring through the RyR pore. Ryanodine binding studies (Meissner et al., 1988), based on binding of the plant alkaloid, ryanodine, which occurs only to the open channel (Fleischer et al., 1985), can also be used as an indirect measure of the RyR channel open probability (*P*_o_), but unlike single channel studies only gives a snapshot of the steady state (Valdivia et al., 1995).

#### 2.2.1 Ca^2+^ sparks

The Ca^2+^-induced Ca^2+^ release (CICR) phenomenon was first proposed as an ECC mechanism in skinned muscle fibres (Fabiato & Fabiato, 1979). Subsequently, fluo-3 confocal microscopy with intact cardiac myocytes enabled observation of spontaneous Ca^2+^ ‘sparks’, which result from simultaneous opening of a group of RyR2s (Cheng et al., 1993). A Ca^2+^ spark can originate from trigger Ca^2+^ (a ‘sparklet’) entering through one LCC (the *I*_Ca_ current) to activate a cluster of opposing RyR2 molecules to discharge SR store Ca^2+^ (Lopez-Lopez et al., 1995; Wang et al., 2001). A study of the quantal nature of Ca^2+^ sparks suggested that normally a few RyR2s were recruited (4–6) although 12% of spark events emanated from a single RyR2 channel opening (Wang et al., 2004). Other studies have estimated greater numbers of RyR2 molecules in a spark cluster; 15–30 (Izu et al., 2001), 10 (Lukyanenko et al., 2000), 18–25 (Lukyanenko et al., 2007) and 35–43 (Mejia-Alvarez et al., 1999). It is also proposed that, in practice a group of LCC may gate a cluster of underlying RyR2s in order to ensure that the probability of a spark from that cluster is sufficiently high to fire reliably with each AP (Inoue & Bridge, 2003). The relationship between the LCC Ca^2+^ current and the quantity of Ca^2+^ released from Ca^2+^ stores is called ‘Ca^2+^ gain’ and defined as the ratio between total Ca^2+^ flux through RyR relative to that through the LCC. The parameters which regulate this process are complex, involving their physical relationship (dyadic cleft), innate regulation of the individual RyR and LCC channels and the numbers activated (Altamirano & Bers, 2007; Wier, 2007).

Many individual sparks coalesce to give the characteristic Ca^2+^ wave/transient, a phenomenon termed the local control or ‘cluster bomb’ hypothesis (Stern, 1992; Rios & Stern, 1997; Williams, 1997; Cannell & Soeller, 1998). The extent of recruitment of RyR2s to a spark depend upon both the mechanisms initiating the spark and the regulatory influences modulating RyR activity, including allosteric interactions of RyRs with their neighbours (Yin & Lai, 2000; Yin et al., 2005a,b) and the disposition of other proteins (Rice et al., 1999). The mechanism of simultaneous activation of the RyR2 molecules within a cluster to produce a spark is unknown. Stochastic gating theory suggests that each RyR molecule activates and closes independently (Klein et al., 1996; Rios & Stern, 1997). However, it has been observed that two or more RyR channels inserted into a bilayer can either gate individually (stochastically) or synchronously (coupled), although the latter was observed only in the presence of the modulator, FK506 binding protein (FKBP12 or 12.6). Coupled activation would require the physical interaction of adjacent RyR molecules (see Section 2.5). Extensive RyR 2D arrays, making up a couplon *in situ* (Franzini-Armstrong et al., 2005; Chen-Izu et al., 2006), can also be formed by purified RyR1 protein (Yin & Lai, 2000; Yin et al., 2005a,b). In the context of ECC, the structural basis of Ca^2+^ sparks and their genesis is incompletely understood (Soeller et al., 2007). 2D arrays of RyR2 could theoretically allow a large population of receptors to be simultaneously switched on, or off, by a tiny change in ligand concentration (Liang et al., 2007; Yin et al., 2008). However, considerations of modal channel gating and Ca^2+^ spark generation involving only a small proportion of RyR molecules within a couplon, raises issues regarding the precise role of RyR oligomeric interaction in ECC (Stern et al., 1999; Fill & Copello, 2002).

The feed-forward and feed-back mechanisms of ECC are relatively well defined with one exception — RyR2 closure (Stern & Cheng, 2004). CICR is a process which is theoretically self-perpetuating (although clearly not so in practice) and thereby considered to be inherently unstable. The activated RyR2 must also close to maintain the fidelity of the heartbeat, but the underlying mechanism(s) leading to closure have been difficult to demonstrate experimentally or definitively. With respect to Ca^2+^ spark termination, the rise time of a spark decreases proportionately with the number of participating RyR2s and it has been suggested that this is due either to inhibitory feed-back by the dyadic Ca^2+^ concentration overriding regenerative CICR (Wang et al., 2004), or to the concomitant decrease in SR luminal Ca^2+^ underlying the spark site (Brochet et al., 2005; Huertas & Smith, 2007). However, there may be other determinants for CICR termination, such as intrinsic changes occurring within the RyR2 such as inactivation, adaptation (Cheng et al., 1995; Sitsapesan et al., 1995; Laver & Lamb, 1998; Fill et al., 2000) and stochastic attrition (Stern et al., 1999) or allostery between RyRs (Yin et al., 2008).

#### 2.2.2 Cytoplasmic Ca^2+^ (dyadic cleft)

Single channel recordings and ryanodine binding experiments have shown that RyR2 open probability, *P*_o_, exhibits a biphasic response to cytoplasmic Ca^2+^ concentration, with an activation threshold of ∼ 10^− 8^ – 10^− 7^ and maximal activation at 10^− 6^ – 10^− 5^ M, that declines with increasing concentrations and maximal inhibition occurring at ∼ 10^− 2^ M Ca^2+^ (Meissner, 2002). Maximal activation is observed in the presence of ∼ 1 mM ATP, in cell free systems, while physiological concentrations of 1 mM Mg^2+^ inhibit *P*_o_ (Meissner & Henderson, 1987; Holmberg & Williams, 1990; Tinker & Williams, 1992), either by competing for Ca^2+^ activation sites, low affinity inhibition sites (Laver et al., 1997) or sites in the Ca^2+^ pore (Valdivia et al., 1995). Ca^2+^ ions that enter the cell upon LCC activation provides ∼ 10% of the Ca^2+^ available for contraction (Bers, 2002).

The small volume of the dyadic cleft and the close proximity of the junctional RyR to LCC results in a rapid local increase in Ca^2+^ concentration, activating RyR2 and evoking systolic Ca^2+^ release from the SR Ca^2+^ store. Models suggest that the dyadic cleft Ca^2+^ concentration can reach 1 mM and the estimated number of Ca^2+^ ions required to achieve this concentration within its defined dimensions (which may be species-dependent) is 10–100 (Tanskanen et al., 2007) or 1000 (Koh et al., 2006) with only 1–2 remaining in diastole (Koh et al., 2006). The concentrations of Mg^2+^ and ATP, which regulate RyR2 and influence the free Ca^2+^ concentration, also undergo dynamic beat to beat changes within the dyad environment (Valent et al., 2007). The released Ca^2+^ diffuses swiftly to the myofilaments, which are in close proximity to the SR Ca^2+^ ATPase (SERCA) pumps and their regulatory protein phospholamban (PLB) situated on the longitudinal SR. SERCA resequesters the released Ca^2+^ into the SR Ca^2+^ store. The raised Ca^2+^ in the dyadic cleft has a retrograde inhibitory action on LCC and also activates the NCX, which effectively drives Ca^2+^ out of the cell (Bers, 2002). Thus, the Ca^2+^ store release by RyR2 is balanced by reuptake through SERCA, and Ca^2+^ influx through LCC is balanced by efflux via the NCX. These coordinated processes of Ca^2+^ ion movement provide a counter balance for each other in order to maintain a steady state (Diaz et al., 2005), Fig. 1.

#### 2.2.3 Luminal Ca^2+^

When CICR was proposed as a RyR activation mechanism it was recognised from the requirement for a threshold, that the amount of Ca^2+^ within the SR exerted a regulatory influence on its release (Fabiato & Fabiato, 1979). Single channel experiments have confirmed that the greater the luminal Ca^2+^ concentration, the more responsive RyR2 becomes to activator Ca^2+^ concentration (in the presence of ATP) (Lukyanenko et al., 1996; Sitsapesan & Williams, 1997; Gyorke & Gyorke, 1998), and a specific Ca^2+^-sensitive luminal regulation site has been proposed (Laver, 2007). This phenomenon has also been observed in cardiomyocytes, where for a given Ca^2+^ trigger, the SR Ca^2+^ release via RyR2 is proportional to the SR Ca^2+^ concentration (Han et al., 1994; Janczewski et al., 1995; Satoh et al., 1997) and spark frequency is also increased with SR Ca^2+^ load (Satoh et al., 1997). Phosphorylation may also play a role in increasing the sensitivity of RyR2 to luminal Ca^2+^ (Xiao et al., 2007a) — see Section 2.4.1.

#### 2.2.4 Calsequestrin, triadin and junctin

It has been proposed that the luminal RyR2 accessory proteins, calsequestrin (CSQ), triadin and junctin, directly regulate the RyR2 channel sensitivity to luminal Ca^2+^ (Gyorke et al., 2004), as part of a process termed luminal Ca^2+^-dependent deactivation (Terentyev et al., 2002; Shannon et al., 2003). CSQ has a low affinity for Ca^2+^ (∼ 600 µM) and each molecule can bind about 40 Ca^2+^ ions, which cause a major conformational change in the protein (Mitchell et al., 1988). Since Ca^2+^ release rises steeply with SR Ca^2+^ concentration, CSQ may therefore play a role in Ca^2+^ buffering within the SR to regulate the luminal free Ca^2+^ concentration (Shannon et al., 2000). In addition, a role for CSQ as the RyR2 luminal Ca^2+^ sensor is also proposed (Beard et al., 2005). The two proteins, triadin (Terentyev et al., 2007) and junctin have luminal and transmembrane regions (Zhang et al., 1997) and form into a complex with CSQ that appears to inhibit RyR *P*_o_ when Ca^2+^ concentration is low and this inhibition is relieved as Ca^2+^ rises (Gyorke et al., 2004).

In adult ventricular rat myocytes, the functional size of the SR Ca^2+^ store was regulated by the amount of CSQ expressed (Terentyev et al., 2003; Miller et al., 2005). In CSQ2 knock-out mice the volume of the SR was doubled, demonstrating a physical compensation for CSQ2 ablation, with expression of junctin and triadin also suppressed via an unknown mechanism (Knollmann et al., 2006). Field-stimulated CSQ2-null myocytes showed a decrease in time to peak Ca^2+^ release and concomitant slowing of the time to 50% peak contraction. In unstimulated myocytes, there was an increase in spontaneous Ca^2+^ sparks (Knollmann et al., 2006) suggesting that the CSQ/triadin/junction complex (Terentyev et al., 2003; Gyorke et al., 2004) might act to reduce the *P*_o_ of RyR2 at high Ca^2+^ load, but that RyR2 could also independently sense Ca^2+^ load in the absence of the CSQ (Knollmann et al., 2006). In addition, at low free Ca^2+^ concentrations the CSQ/triadin/junction complex may be inhibiting RyR2 directly. Luminal Ca^2+^-dependent deactivation has been proposed to provide a mechanism for termination of CICR (Gyorke & Terentyev, 2008). In support of this mechanism one study has demonstrated, with sufficient spatial and temporal resolution, a decrease in luminal Ca^2+^ concentration (termed a Ca^2+^ ‘blink’), immediately beneath a RyR2 cluster during Ca^2+^ release from the SR, which complemented a Ca^2+^ spark in the dyadic space (Brochet et al., 2005).

### 2.3 Ryanodine receptor type-2 and the generation of delayed after depolarisations

As well as the maintenance of Ca^2+^ homeostasis, the dyadic cleft is vitally important to maintaining the electrical coupling which regulates the heart-beat. In response to the AP, LCC is activated and Ca^2+^ moves down the diffusion gradient into the dyadic cleft. For the heart to relax the cell must repolarise. The LCC is sensitive to retrograde inhibition by the rate and quantity of Ca^2+^ released by RyR2 from the SR Ca^2+^ store and is also shut off by repolarisation of the membrane by K^+^ channels (Richard et al., 2006). The Na^+^/K^+^ ATPase and NCX also play a role in this process, thus dovetailing the electrogenic exchange with the maintenance of Ca^2+^ homeostasis (Scoote & Williams, 2002; Pogwizd & Bers, 2004).

**Fig. 2:**
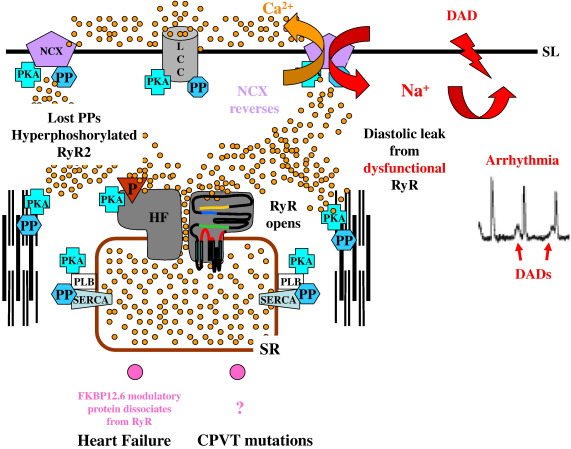
Relationship between diastolic RyR2 Ca^2+^ leak, mechanisms of RyR2 dysfunction and the generation of DADs. A number of causes of diastolic leak from dysfunctional RyR2 are proposed for HF. These include ‘hyperphosphorylation’ of RyR2 and altered sensitivity to Ca^2+^. In CPVT mutations, perturbation of RyR2 domain interactions, and increased RyR2 sensitivity to luminal Ca^2+^, have been proposed. The consequent increase in released Ca^2+^ is thought to trigger the NCX to cause an influx of Na^+^ that leads to a compensatory transient DAD. See the text for further explanation.

The type of arrhythmia which is the topic of this review involves DADs which originate from within the myocyte and are blocked by ryanodine (Marban et al., 1986; Song & Belardinelli, 1994). These DADs are caused by an inappropriately timed, spontaneous efflux of Ca^2+^ escaping the SR via the RyR2, termed a diastolic ‘leak’ (see Fig. 2). Note that this is not the SR Ca^2+^ flux that can be measured through reversal of SERCA (Shannon et al., 2000) which has also been called diastolic Ca^2+^ leak. If sufficient Ca^2+^ is released into the dyadic space after repolarisation but before the next AP, this will cause NCX to operate in reverse (Na^+^ entry mode). This NCX activity leads to a depolarisation which invokes the transient inward current — *I*_ti_ (Venetucci et al., 2007), that propagates to adjacent cells provoking an arrhythmia (for a more detailed consideration see Scoote & Williams, 2002; Pogwizd & Bers, 2004).

DADs can also be caused by SR Ca^2+^ store overload (Diaz et al., 2004) and by the action of glycosides on the Na^+^/K^+^ ATPase (Rosen & Danilo, 1980; Wier & Hess, 1984; Hauptman & Kelly, 1999). Increasing the *P*_o_ of RyR2 by addition of low caffeine concentrations alone increases Ca^2+^ spark frequency in cardiomyocytes, but these are of low amplitude due to decrease in SR Ca^2+^ stores (Trafford et al., 2000), and do not produce DADs unless the myocytes are subjected to β-adrenergic stimulation (Venetucci et al., 2007). Caffeine also produces DADs in ventricular wedge preparations (Nam et al., 2005) under similar circumstances. Thus, increasing RyR2 *P*_o_ *per se* is not arrhythmic, since this would be traded against decreased store content (Aizawa et al., 2007; Venetucci et al., 2007). The effect of β-adrenergic stimulation is to increase SR Ca^2+^ store concentrations via phosphorylation of PLB and upregulation of SERCA. However, phosphorylation may also alter RyR2 channel gating through direct phosphorylation of the RyR2 (see Section 2.4) or through regulation by luminal Ca^2+^, thus suggesting that perturbing both autoregulation of RyR2 and Ca^2+^ homeostasis may be necessary to provoke DADs (Niggli, 2007). Conversely, DADs triggered by such a combined perturbation could be reduced by tetracaine, which reduces the *P*_o_ of RyR2 (Venetucci et al., 2006).

### 2.4 Phosphorylation/dephosphorylation

**Fig. 3:**
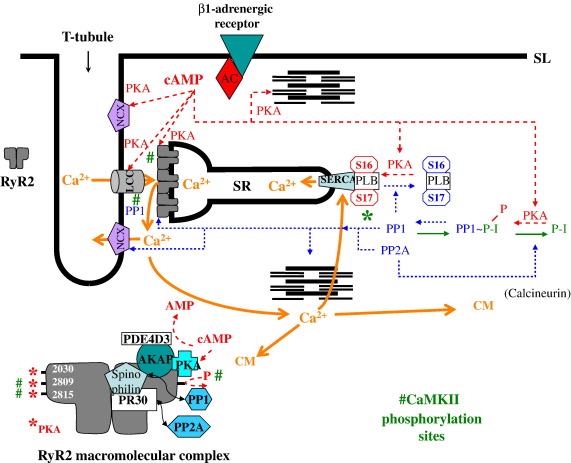
Regulation of ECC by phosphorylation/dephosphorylation. In response to β-adrenergic receptor stimulation by circulating catecholamines, adenyl cylase (AC) is activated and the diffusible second messenger cAMP is produced. This activates PKA which is associated with, and phosphorylates, a large number of proteins (NCX, LCC, RyR2 and PLB). PKA interacts with these target often via the scaffolding protein, AKAP. The key PKA targets are indicated by dashed lines. The action of PKA increases the activity of these target proteins and augments the rate of Ca^2+^ cycling in ECC, leading to an elevated heart rate. CaMKII is activated by Ca^2+^ (via calmodulin; CM) and can also activate key proteins in ECC and these are indicated by #. Protein phosphatases down-regulate ECC by dephosphorylation of the kinase target proteins (indicated by dotted lines), and can in turn be regulated by P-I. The major directions of Ca^2+^ ion ‘movement’ and the additional regulation of ECC by Ca^2+/^CM are indicated by the solid lines. The lower left inset schematically depicts the RyR2 macromolecular complex that has been proposed to exist by Marks and colleagues, illustrating its association with those proteins concerned with phosphorylation/dephosphorylation. Several other cytosolic proteins are known to interact with RyR2, although their precise functional role remains to be clarified. The inset also indicates the mouse RyR2 amino acid number of the three known serine phosphorylation sites that are associated with PKA (⁎) and CaMKII (#) action. Further explanation is given in the text.

Kinase/phosphatase signalling complexes have been described for three cardiac ion channels; LCC, K^+^ channels and RyR2, created by the association of phosphatases and kinases with anchoring proteins held together by leucine zipper protein/protein interaction domains (Marx et al., 2001b; Hulme et al., 2004). These complexes enable the compartmentalisation of protein kinase A (PKA), protein phosphatase 1 (PPI), protein phosphatase 2A (PP2A) (Marx et al., 2001b), CaMKII (Ai et al., 2005), and phosphodiesterase 4D3 (PDE4D3) (Dodge et al., 2001), so that phosphorylation/dephosphorylation can be spatially and temporally regulated (Hulme et al., 2004). For the RyR2 complex, PKA and PDE4D3, are reported to be organised via the scaffold protein, muscle-specific A-kinase anchoring protein (mAKAP) (Dodge et al., 2001), and PP1 and PP2A associate with RyR2 via the anchoring proteins spinophilin and PR130, respectively (Marx et al., 2001b) (see Fig. 3). RyR2 currently has three well-defined phosphorylation sites, S2030 (Xiao et al., 2005), S2809 and S2815 although there may be others (Rodriguez et al., 2003). S2030 and S2809 are phosphorylated by PKA, and S2809 (Witcher et al., 1991; Marx et al., 2000b; Rodriguez et al., 2003) and S2815 by Ca^2+^/calmodulin-dependent protein kinase II (CaMKII) (Witcher et al., 1991; Wehrens et al., 2004b).

#### 2.4.1 Phosphorylation by protein kinase A

The flight-or-fight response, driven by adrenergic stimulation of cardiac β-adrenoreceptors raises intra-myocyte cAMP, which causes PKA activation. PKA can phosphorylate and co-ordinately regulate (either directly, or indirectly via a regulatory protein) all of the major components of the ECC cycle (see Bers, 2002 for review, also Fig. 3). This has the effect of increasing the heart rate and the force of contraction (Lakatta, 2004).

The effect of phosphorylation on RyR2 activity in myocytes has been difficult to define experimentally. PKA phosphorylation has been reported to increase the synchronicity of Ca^2+^ sparks (Song et al., 2001) and RyR2 coupling fidelity (probability of activating a release site; Shen, 2006), increase the amplitude of Ca^2+^ spark width and duration (Tanaka et al., 1997; Song et al., 2001; Shen, 2006), or to have little effect (Hussain & Orchard, 1997). Ca^2+^ gain or fractional Ca^2+^ release, a measure of Ca^2+^ release/*I*_Ca_, is similarly reported to be enhanced (Hussain & Orchard, 1997), unchanged (Ginsburg & Bers, 2004) or reduced by phosphorylation (Song et al., 2001). Upregulation of Ca^2+^ release could occur as a result of (i) phosphorylation of LCC enhancing *I*_Ca_ (Yue et al., 1990; Herzig et al., 1993; Chen-Izu et al., 2000), (ii) phosphorylation of PLB at S16 or T17 (deinhibiting and upregulating SERCA (Li et al., 2000)) thus increasing SR store Ca^2+^, (iii) an increased sensitivity of phosphorylated RyR2 to activator Ca^2+^ concentrations (Marx et al., 2000b), or (iv) an increased sensitivity to luminal Ca^2+^ concentrations (Xiao et al., 2007a). Experiments that manipulate the Ca^2+^ store using thapsigargin (SERCA inhibitor) have shown Ca^2+^ release to be governed by *I*_Ca_ (Hussain & Orchard, 1997) such that release is graded by increased trigger Ca^2+^. Others have suggested that the size of the Ca^2+^ store governs increased Ca^2+^ release. PLB knock-out or PLB-DM (double-mutant) knock-in mice with S16A/T17A substitutions, in which the SR Ca^2+^ store would remain constant, have shown that in permeabilised myocytes (no *I*_Ca_), isoprenaline did not alter Ca^2+^ sparks (Li et al., 2002). A second study with the PLB-DM mice showed that fractional Ca^2+^ release over a wide range of *I*_Ca_ and SR Ca^2+^ store concentrations was unchanged by isoprenaline, suggesting that although the overall balance of influx and release was maintained, Ca^2+^ release was turned on and off more quickly with isoprenaline treatment and these faster kinetics were attributed to the effect of RyR2 phosphorylation (Ginsburg & Bers, 2004).

Single channel studies of RyR2 channel gating have shown that PKA phosphorylation can overcome Mg^2+^ block (Hain et al., 1995) rendering the channel active at physiological Mg^2+^ concentrations (∼ 1 mM), while ATP no longer modified channel gating though *P*_o_ was increased (Valdivia et al., 1995; Uehara et al., 2002). PKA phosphorylation decreased ryanodine binding, and RyR2 channel opening became more responsive to a stepped increase in Ca^2+^, although the rate of channel adaptation was also increased (Valdivia et al., 1995). This might result in lower steady state activity which manifests as a decrease in ryanodine binding (Valdivia et al., 1995), consistent with a study in cardiomyocytes demonstrating an increased rate of channel opening matched by an increased rate of termination in the presence of isoprenaline (Ginsburg & Bers, 2004). Analysis of sheep RyR2 suggests that in normal conditions there is already a substantial level of phosphorylation (S2809 phosphorylated to 75%) (Carter et al., 2006). Stoichiometric phosphorylation of S2809 with PKA resulted in increased conductance and increased *P*_o_. However, since PKA can phosphorylate RyR2 at two sites, S2809 and S2030, PKA-dependent phosphorylation of S2030 may specifically be responsible for the observed functional effects on single RyR2 channels (Xiao et al., 2005). Interestingly, RyR2 dephosphorylation also caused a modest increase in *P*_o_ (Carter et al., 2006). Others have reported that PKA phosphorylation increased *P*_o_ and produced subconductance states (presumably related to the loss of endogenous FKBP12.6, see HF in Section 4.1.2.2.) and it was proposed that phosphorylation increased the Ca^2+^ sensitivity of activation (Marx et al., 2000b). However, another phosphorylation study observed no change in activation by cytosolic (cis) Ca^2+^ on single channel analysis, but specifically linked S2030 phosphorylation to an enhanced response of RyR2 to luminal (trans) Ca^2+^ (Xiao et al., 2007a).

#### 2.4.2 Phosphorylation by Ca^2+^-calmodulin kinase II

A second protein kinase, CaMKII (the *δ*_C_ isoform in heart (Zhang et al., 2007)) is activated by [Ca^2+^]_i_ possibly at high cellular Ca^2+^ loads (MacQuaide et al., 2007) and is associated with the phosphorylation of many of the same ECC proteins as PKA. Phosphorylation of LCC can enhance *I*_Ca_ (Wu et al., 2004). Experimentally, phosphorylation of PLB at T17 occurs in a frequency-dependent manner in electrically-paced myocytes and the increased cytosolic Ca^2+^ causes autophosphorylation of CaMKII, which is proposed to sustain phosphorylation levels of PLB T17 between beats (Hagemann et al., 2000) thus increasing both the rate of SERCA and SR store Ca^2+^ levels (Hagemann et al., 2000). *In situ* increased frequency of beating results from the activation of PKA through β-adrenergic stimulation, which in turn increases cytosolic Ca^2+^ hence activating CaMKII. Thus, distinguishing their precise roles and the relative importance of phosphorylation of RyR2 by PKA and CaMKII is problematic (Bers, 2006; Sipido, 2007; Yamaguchi & Meissner, 2007).

Perfusing rat hearts with isoproterenol, to activate PKA, upregulated the CaMKII pathway and both RyR2 S2808 (PKA) and S2815 (the phosphorylation site for CAMKII (Witcher et al., 1991; Wehrens et al., 2004b)) were phosphorylated (Ferrero et al., 2007). In another study, the use of specific CaMKII inhibitors or using forskolin to bypass β-adrenergic stimulation and activating adenyl cyclase directly, was consistent with increased diastolic Ca^2+^ leak being mediated by CaMKII but not PKA (Curran et al., 2007). Two studies have used the CaMKII inhibitory peptide AIP, to reduce the activity of endogenous CaMKII. In rabbit cardiomyocytes, AIP caused decreased ryanodine binding and Ca^2+^ spark activity (Currie & Smith, 1999). Mice expressing a SR targeted AIP showed a 62% reduction in RyR2 S2815 phosphorylation and decreases in SR Ca^2+^ release via RyR, SR Ca^2+^ uptake (SERCA activity) and Ca^2+^ entry via LCC (Picht et al., 2007). Both these studies therefore support the view that endogenous CAMKII activity increases RyR *P*_o_, SR Ca^2+^ release via PLB regulation of SERCA and LCC activity. However, Wu et al. used both constitutively active CAMKII and an inhibitory peptide AC3-I in ventricular myocytes to show that although LCC activity is increased, RyR2 activity is reduced by CaMKII addition (Wu et al., 2001). Using a viral expression system the effects of constitutively active CaMKII, WT and inactive CaMKII were compared in rat myocytes. PLB phosphorylation was increased by the constitutively active isoform and the global decay of the Ca^2+^ transient was increased and store Ca^2+^ rose, consistent with upregulation of SERCA. However Ca^2+^ sparks and Ca^2+^ transients were suppressed, indicative of a decrease in spontaneous Ca^2+^ release and a negative regulation of RyR2 activity. This was proposed as a mechanism that restrained the positive feedback effects of raised Ca^2+^ and thus stabilised ECC (Yang et al., 2007).

Single channel studies of the effects of CaMKII phosphorylation of RyR2 also give inconsistent results. Some reported observing similar characteristics to PKA phosphorylation, in that CaMKII phosphorylation was able to reverse Mg^2+^ inhibition in single channel studies, however, these showed that *P*_o_ was decreased overall (Wang & Best, 1992; Hain et al., 1995; Lokuta et al., 1995) and ryanodine binding studies also revealed a reduced *P*_o_ (Lokuta et al., 1995). Others found that CaMKII phosphorylation increases *P*_o_ of RyR2 which is specifically related to the S2815 phosphorylation site (Wehrens et al., 2004b).

Thus, although phosphorylation is generally reported to upregulate the ECC cycle there is a disconcerting lack of consistency in the results of many studies, particularly concerning the mechanism of action of phosphorylation on RyR2 function and whether effects are mediated by PKA or CaMKII (Bers, 2006; Sipido, 2007; Yamaguchi & Meissner, 2007).

#### 2.4.3 Dephosphorylation

To counter up-regulation by phosphorylation are the equally important processes which return ECC to the *status quo ante* by dephosphorylating the component proteins when the stimulus diminishes. Dephosphorylation is carried out by protein phosphatases and isoforms PP1, PP2A (Neumann et al., 1993) and calcineurin are all expressed in heart (Jones et al., 2003). Binding domains on RyR2 have been identified for PP1 and PP2A (via scaffolding proteins) (Marx et al., 2001b). The protein phosphatases PP1 and PP2A also affect SERCA activity by dephosphorylation of PLB (MacDougall et al., 1991). To add a further layer of complexity PPI can itself be phosphorylated by PKA, which inhibits its action and dephosphorylation of PP1 (by PP2A) can be inhibited by the action of phosphatase-inhibitors-1 or -2 protein (P-1/2) (Endo et al., 1996), Fig. 3.

### 2.5 Regulation of ryanodine receptor type-2 by FKBP12.6

FKBP12 modulates RyR1 and the FKBP12.6 isoform modulates RyR2. These 12 kDa proteins are thought to be potent and important regulators of RyR function. They bind with high affinity in both the open or closed state of the channel although the affinity is greater when the channel is closed (Jones et al., 2005b) and indeed interaction with the FKBP12 protein was first identified by its persistent co-purification with RyR1 channels (Collins, 1991; Jayaraman et al., 1992). One FKBP12 interacts with one RyR1 subunit (Jayaraman et al., 1992; Timerman et al., 1995) and topological analysis has shown four FKBP12 molecules bound symmetrically proximal to the N-terminus (Wagenknecht et al., 1997). The binding site on RyR was originally thought to be in the central domain around leucine-proline site 2407–2520 (RyR1) (Cameron et al., 1997) or 2361–2496 (RyR2) (Marx et al., 2000), which are homologous to the proposed binding region on the inositol (1,4,5) tris phosphate receptor (Cameron et al., 1997). Studies mutating key residues in this region have shown these to decrease FKBP12/12.6 binding (Bultynck et al., 2001b; Gaburjakova et al., 2001; Avila et al., 2003; Van Acker et al., 2004). The use of limited proteolysis of RyR2, mapped the 2756–2803 epitope — recognised by the m34C antibody, to within a 45 kDa FKBP12.6 binding fragment (Bultynck et al., 2001a). One study showed binding to the 2341–2356 RyR2 site using the yeast-2-hybrid technique (Marx et al., 2000) although another study failed to pick out this region using the same technique or recombinant expression of overlapping RyR2 fragments and FKBP12.6 pull-down assays (Zissimopoulos & Lai, 2005a). In topology studies the central domain cluster (2246–2534) was mapped to the N-terminus region, which was not consistent with the site mapped for FKBP12 binding to RyR1 (Wagenknecht et al., 1997). Others have proposed a different binding site using recombinant constructs of the RyR sequence — one in the N-terminus (305–1937) (Masumiya et al., 2003) and another at the C-terminus potentially involving VPLV (4594–4597 hRyR2) (Zissimopoulos & Lai, 2005a). Thus to date the region of RyR1/2 binding to FKBP12/12.6 has defied precise definition.

Some studies have shown that FKBP12/12.6 act to stabilise RyR1/2 and inhibit channel activity reducing *P*_o_ in bilayer studies (Brillantes et al., 1994; Chen et al., 1994; Ma et al., 1995). These effects were reversed by FK506, which binds to FKBP12/12.6 at its RyR binding site and inhibits interaction (Chelu et al., 2004). The result was to increase *P*_o_ and in some experiments sub-conductance states were seen leading to the conclusion that FKBP12/12.6 binding might stabilise RyR inter subunit interactions (Brillantes et al., 1994; Ahern et al., 1997).

A second action of FKBP12/12.6 may be to modulate, functionally but not structurally, coupled gating (Marx et al., 1998; Marx et al., 2001a). *In situ* RyRs are organised in 2D arrays (Franzini-Armstrong et al., 2005) and in this 2D arrangement the binding domain for FKBP12 is distinct from the RyR–RyR interacting domains 6 (Wagenknecht et al., 1997; Yin et al., 2005a) and divergent region 2 (Liu et al., 2004). A study of RyR1 oligomeric interaction using photon correlation spectroscopy showed no difference in association with or without added FKBP12 (Hu et al., 2005).

FKBP12.6 overexpression resulted in a decreased number of spontaneous Ca^2+^ sparks which were of decreased amplitude, width and duration in both mouse (Gomez et al., 2004) and rabbit cardiomyocytes (Loughrey et al., 2004). This was interpreted as an effect on decreasing diastolic Ca^2+^ leak particularly as this was accompanied by an increase in the SR Ca^2+^ store content (Gomez et al., 2004). However, the Ca^2+^ transient amplitude was increased, which accounted for the observed increases in contractility and thus the equivalent of improved systolic function was seen (Prestle et al., 2001; Gomez et al., 2004). A similar increase in store Ca^2+^ and an increased Ca^2+^ transient were observed in rabbit cardiomyocytes (Loughrey et al., 2004). It was concluded that the changed dynamics of Ca^2+^ release may result from improved coupled gating and early termination of the spark by FKBP12.6 (Gomez et al., 2004; Loughrey et al., 2004). Generally the effects of FK506 on cardiomyocytes were the reverse of FKBP12.6 overexpression. Differences were observed, however, between rat and rabbit cardiomyocytes. The latter showed a decrease in SR store Ca^2+^ which was attributed to the greater reliance on NCX extrusion of released Ca^2+^ in rabbit decreasing dyadic Ca^2+^ concentration, despite increased Ca^2+^ diastolic leak caused by the FK506 removal of stabilising FKBP12.6 (Su et al., 2003). FKBP12 can also bind to RyR2 (Jeyakumar et al., 1998) but co-expression with RyR2 in CHO cells did not functionally modulate the channel, whereas FKBP12.6 expression rescued these cells from loss of viability and abnormalities of Ca^2+^ signalling (George et al., 2003). FKBP12 over expression in rabbit ventricular myocytes, however, modulated ECC in a manner which suggested that it reduced RyR2 sensitivity to Ca^2+^ (Seidler et al., 2007) but was different from overexpression of FKBP12.6 (Loughrey et al., 2004).

Two models of FKBP12.6 knockout mice have been produced in different strains, which show different characteristics. In one the males, but not females, developed hypertrophy and the mice showed evidence of perturbed Ca^2+^ signalling ‘at rest’ as CICR gain was increased manifest as an increase in amplitude and duration of spontaneous Ca^2+^ sparks (Xin et al., 2002). In the second model there was no evidence of hypertrophy and at rest Ca^2+^ signalling was similar to that of WT mice however, when the hearts were paced or given an isoprenaline injection arrythmogenic beats were observed (Lehnart et al., 2006), in contrast to the other model, which showed no arrhythmic tendencies when challenged with caffeine plus epinephrine (Xiao et al., 2007b). It has been suggested that the surprisingly moderate effects of FKBP12.6 ablation on ECC (and RyR2 in particular) might be attributable to surrogate regulation by FKBP12 in these animals (Seidler et al., 2007).

The importance of regulation by FKBP12/12.6 is therefore contentious and particularly relevant to the proposed mechanisms of RyR2 dysfunction in HF and CPVT (see Sections 4.1.2.2 and 4.2.3.2).

## 3 Ryanodine receptor molecular structure

**Fig. 4:**
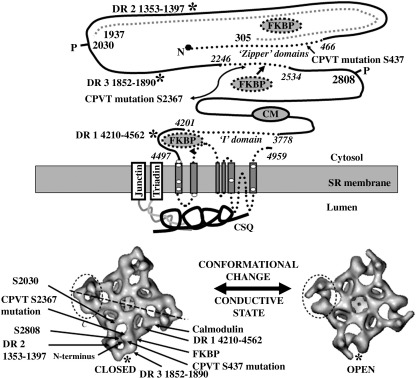
RyR2 structure and the relationship to important domains associated with function, regulatory proteins and CPVT point mutations. The upper diagram is a schematic illustration of the RyR2 polypeptide sequence with the proposed locations of modulatory protein binding sites for FKBP12.6 (dotted grey oval with arrows) (Marx et al., 2000; Masumiya et al., 2003; Zissimopoulos & Lai, 2005b) and for CM (grey oval above the transmembrane domain). CPVT mutation clusters are illustrated within the RyR2 polypeptide as dotted lines, with amino acid numbers in italics indicating their boundaries (residues 1–466, 2246–2534, 3778–4201, 4497–4959). Divergent regions identified within the RyR2 sequence numbering given (DR 1, DR 2 or DR 3) are shown by an asterisk (Liu et al., 2002; Zhang et al., 2003a; Liu et al., 2004). The position of CPVT mutation S437 (Wang et al., 2007) and S2367 (Liu et al., 2005), which have been mapped to the corner region of RyR2 (see below) by insertion of GFP into the RyR2 sequence are shown. Two PKA phosphorylation sites, P2808 (Meng et al., 2007) and P2030 (Jones et al., 2007) are both marked and have also been mapped. The lower figure is a topology model of RyR1 structure, viewed from the cytosol (modified from Serysheva et al., 1999; Fig. 3, with permission) depicting the open channel conformation (right panel; 100 µM Ca^2+^ plus AMP–PCP) compared to the closed channel (left panel; EGTA). Noted by an asterisk is the significant change in each corner of the structure apparent when comparing the closed versus open RyR conformation. Indicated by arrows on the closed conformation, are the proposed locations of some of the domains mentioned above; PKA S2030 phosphorylation site (Jones et al., 2007), PKA S2809 phosphorylation site (Meng et al., 2007) the N-terminus (Liu et al., 2001), S437-GFP (Wang et al., 2007), DR1 (Liu et al., 2002), DR2 (Liu et al., 2004), DR3 (Zhang et al., 2003a), S2367-GFP (Liu et al., 2005) and the proposed binding locations of FKBP12 and calmodulin (Wagenknecht et al., 1997). These sites are illustrated with greater precision in the original papers, and these also show their locations in other viewing angles, but their clustered distribution illustrated here suggests that the RyR corner region (encircled with dotted line) may be important in regulation of the conformational changes that mediate channel gating. Note also the apparent close proximity of several distant regions of the RyR linear sequence suggesting significant contribution of protein folding to the overall RyR channel structure.

RyR is a homotetramer with a central Ca^2+^ pore and each subunit has a molecular mass of ∼ 560 kDa making RyR the largest known Ca^2+^ channel protein with a molecular mass of ∼ 2200 kDa (Lai et al., 1988). The homotetramer is extremely stable and the purified protein, because of its enormous size, is amenable to cryo-electron microscopy and image analysis to produce electron density maps of the molecule (Samso & Wagenknecht, 1998; Serysheva, 2004) at 14 A (Serysheva et al., 2005) and ∼ 30 A (Radermacher et al., 1994; Sharma et al., 1998). These topology models have revealed a tetragonal structure with a large cytoplasmic region and a transmembrane ‘stalk’ — see Fig. 4. C terminus constructs of RyR2 expressed in CHO cells (3550–4976 (Bhat et al., 1997), 3722–4967 and 4485–4967 (George et al., 2004)) all formed tetrameric Ca2^+^ channels, albeit poorly regulated. The extreme C terminus 100 amino acids of RyR2 were able to self tetramerise (Stewart et al., 2003) and the mutation of key charged residues within this region of RyR1 suggested that electrostatic interactions may contribute to tetramerisation (Lee & Allen, 2007). Deletion of the final 15 amino acids from a full length construct of RyR1 impaired tetramer formation (Gao et al., 1997). Sequence analysis of the RyR protein shows that the C terminus sequence (the last 20% of the full length) contains up to 12 hydrophobic helical regions, which could be the transmembrane domains (Takeshima et al., 1989; Nakai et al., 1990; Otsu et al., 1990; Zorzato et al., 1990; Hakamata et al., 1992; Tunwell et al., 1996). Models suggest 4 (Takeshima et al., 1989), 6 (Tunwell et al., 1996), 12 (Zorzato et al., 1990) or 6–8 transmembrane regions per monomer with one potential transmembrane stretch forming a pore loop from the luminal side (Du et al., 2002). The latter bears sequence homology to the K^+^ channel pore loop and mutations in this loop of RyR2 had severe consequences on conductance and ryanodine binding (Zhao et al., 1999). Comparative modelling of the extreme RyR2 C-terminus loops, with (KcsA) K^+^ channel crystal structure (Doyle et al., 1998), revealed equivalent arrangement and structure (Welch et al., 2004). A high resolution cryo-EM study at ∼ 10 A (of the closed RyR1 molecule) revealed a transmembrane substructure similar to that of the K^+^ channel (KscA) for the inner helix of each monomer but a poor fit for the outer helices (Samso et al., 2005). A second high resolution cryo EM study of the closed RyR1 molecule (Ludtke et al., 2005) compared the projected helical densities with K^+^ channel structures and found a closest structural fit to the MthK channel (Jiang et al., 2002c). The precise number of transmembrane loops is unknown but such knowledge may be very important to understanding the internal RyR molecular interactions which gate the channel between its open and closed configurations.

The topology models show that the RyR1/RyR2 molecule undergoes domain rearrangement(s) during the open to closed conductive transition (Orlova et al., 1996; Serysheva et al., 1999) and RyR2 has a different conformation when bound to FKBP12.6 (Sharma et al., 2006). There is evidence from mapping that the N terminus (Liu et al., 2001), the N-terminus CPVT mutational cluster of RyR2 (amino acids 414–466, GFP insertion at S437 (Wang et al., 2007)) and central domain region containing CPVT point mutations (GFP insertion at 2367) (Liu et al., 2005) are located at the corners of the molecule in the clamp regions (see Fig. 4). The many domains which map to this corner of the molecule, when compared to the sequence of RyR, demonstrate how the tertiary structure of the protein may bring together quite distant regions of the protein sequence by convoluted folding. If, as shown by the difference in the closed and open states, the N-terminus is a domain that is particularly susceptible to conformational change it maybe vulnerable to molecular rearrangements that actually originate elsewhere in the molecule such as an allosteric shift caused by the binding of a modulatory protein or the insertion of a tag such as GFP.

### 3.1 Conformational change and ryanodine receptor domain interaction

One of the unknowns in RyR channel gating is how the four subunits of RyR work together and the domain interactions discussed below may be intrasubunit or intersubunit in origin. The RyR molecule is extremely stable in that purified protein maintains its homotetrameric structure in the presence of mild detergents (Lai et al., 1989). The C-terminus domain has been identified as a necessary requirement for tetramerisation (Gao et al., 1997; Stewart et al., 2003). The importance of interdomain interactions were illustrated by crosslinking studies (Wu et al., 1997). The involvement of the N-terminal region to domain interaction was highlighted by the action of a monoclonal antibody to the G341 region of RyR1, which increased the Ca^2+^ sensitivity and ryanodine binding of SR vesicles and inhibited interaction in overlay assays with domains 799–1172 and 3010–3225 (Zorzato et al., 1996) the latter having been identified as a calmodulin binding domain (Menegazzi et al., 1994). Peptides to the N-terminus domain were shown to alter ryanodine binding and SR Ca^2+^ release in cardiac and skeletal muscle microsomes. It was concluded that the peptide could insinuate between the interacting domains and disrupt interaction by binding to the partner domain (El-Hayek et al., 1999). The synthetic peptide DP4 (2442–2477 of RyR1) was able to disrupt a proposed N-terminus/central zipper domain interaction, Fig. 4, (Yamamoto et al., 2000) and was able to increase Ca^2+^ spark frequency in permeabilised frog skeletal muscle fibres (Shtifman et al., 2002). A similar peptide was produced for RyR2 studies DPc10 (G2460–P2495) and increased the sensitivity of RyR2 to activating Ca^2+^ and increased ryanodine binding (Yamamoto & Ikemoto, 2002).

Another interdomain interaction is proposed between the two C-terminus regions 3534–4610 and 4497–4959 (George et al., 2004). The latter correspond to the sequence postulated to contain the transmembrane segments according to the six transmembrane model (Williams et al., 2001; Du et al., 2002), which surround the channel pore. The 3722–4610 sequence consists of regions of hydrophobic domains. When it was expressed as an independent tagged peptide it interacted with and regulated the C-terminal pore of RyR2 stably expressed in CHO cells (George et al., 2004). The 3534–4610 sequence was termed the I-domain and is rich in residues, domains and motifs implicated in channel regulation (George et al., 2006). Many of these have been characterised in RyR1 or RyR3 but are conserved and have homologous regions in RyR2. The Ca^2+^ sensor residue was identified in RyR3 but the homologous residue in RyR2 is E3988 — this charged residue was mutated to alanine resulting in a 10,000-fold decrease in Ca^2+^ sensitivity (Chen et al., 1998). 4-chloro-m-cresol, which like caffeine is used as a pharmacological activator of RyR channels, required residues Q4020 and K4021 to activate RyR1 (Fessenden et al., 2006). The 3778–4201 domain is also proposed (in RyR1) to contain a CaM-like binding domain 3534–4610, which may interact with the CaM binding site in the 3614–3643 region (Xiong et al., 2006). These domain interactions are proposed to maintain the channel in its closed state but undergo a conformational change as part of the molecular rearrangement of the RyR as it gates from the closed to open conductive state.

The tetragonal symmetry of the RyR provides four identical regulatory sites for every RyR modification or interaction. It is unknown whether they require saturation (i.e all sites to be occupied) or whether there is any co-operativity once one subunit is modified. In this respect it has been calculated that the stoichiometry for RyR2 and FKBP12.6 is > 1:3 (Timerman et al., 1996). It has been proposed that RyR2 from sheep heart is normally 75% phosphorylated (Carter et al., 2006). It is also not known whether the postulated interdomain contacts e.g. between zipper domains, is internal to one subunit or involves inter-subunit interaction. Even with one interaction and four sites it is calculated that there are 54 combinations in which a single modulator can interact with a tetrameric molecule (Bray & Duke, 2004). With so many modulators, including interacting with itself in an array (Yin et al., 2005a), the potential heterogeneity of modulation, which can be experienced by a single RyR molecule reaches theoretically mind-numbing proportions. Yet there is no evidence that this usually causes the RyR any functional difficulty and how the RyR actually overcomes this theoretical problem, and whether it impacts on function in pathological circumstances, is unknown and experimentally difficult to address.

## 4 Ryanodine receptor type-2 and the pathophysiology of fatal arrhythmia

The dysfunction of RyR2 resulting in Ca^2+^ diastolic leak causing DADs is a feature of fatal arrhythmia in HF and CPVT. Patients with the latter condition carry point mutations in the RyR2 gene. In CPVT arrhythmias are triggered by exercise or emotional stress implicating adrenergic drive (Priori et al., 2002). In HF persistent circulating levels of catecholamines are associated with disease progression (Lohse et al., 2003; Molenaar & Parsonage, 2005). Thus both conditions are causally linked to mechanisms involving phosphorylation. The role of RyR2 in the pathophysiology of HF and CPVT will be discussed in the context of its role in ECC, and the mechanisms underlying dysfunction and how these might be changed by phosphorylation, RyR2 interaction with regulatory proteins, Ca^2+^ homeostasis or abnormal intra RyR2 domain interactions.

### 4.1 Heart failure

In Britain 750,000 people are living with HF (www.bhf.org.uk) and statistics suggest that 50% will eventually die from SCD associated with an arrhythmic episode, usually a ventricular tachycardia (VT) leading to ventricular fibrillation (Tomaselli & Zipes, 2004). HF is a disease of complex aetiology, originating from diverse causes with different rates and phenotypic pathways of progression (Hasenfuss & Pieske, 2002; Sipido & Eisner, 2005; Swynghedauw, 2006) raising the question as to whether these are all likely to converge to a common mechanism causing VT and SCD (Pogwizd & Bers, 2004; Tomaselli & Zipes, 2004).

There are a number of defining features of HF. The human myocardium shows depressed contractility and a negative force frequency relationship — essentially as the heart rate increases the failing heart becomes more distressed (Hermann et al., 1999; Hasenfuss et al., 2002). There is compelling evidence at the myocyte level that the progression to HF eventually results in defects in ECC, most specifically changes in Ca^2+^ homeostasis and a depressed level of the SR Ca^2+^ store (Hermann et al., 1999; Belevych et al., 2007) and the resultant decreased release Ca^2+^ from the SR at each beat becomes insufficient to fully activate the myofilaments. This scenario is observed in both experiments from animal models (Eising et al., 1994; Hobai & O'Rourke, 2001; Jiang et al., 2002b; Belevych et al., 2007) and on tissue from human myocardium obtained from transplant patients (Gwathmey et al., 1987; Hasenfuss et al., 1994; Beuckelmann et al., 1995; Hermann et al., 1999; Pogwizd et al., 2001; Jiang et al., 2002b; Piacentino et al., 2003). Reduced SR Ca^2+^ content can arise by a decrease in SERCA expression or degree of activation by PLB or through increased diastolic leak via RyR2 (Bers et al., 2003; Belevych et al., 2007).

The changes characteristic of HF, that are outlines briefly above, can result in arrhythmia but the causative molecular mechanisms are controversial. Global changes in ECC can, because of RyR2's pivotal position, cause dysfunctional changes in RyR2 activity that are difficult to disentangle from innate alterations in the RyR2 protein. The latter includes direct modifications such as phosphorylation by PKA and/or CaMKII and oxidation. There are also potential changes in the stoichiometry with regulatory proteins to consider and loss of FKBP12.6 has been the object of specific focus. Variations in the dynamics and/or concentration of activating or luminal Ca^2+^ may be caused by functional changes in other Ca^2+^ pumps, ion channels or exchangers and influence RyR2 channel gating.

In addition to RyR2-centred mechanisms, there are other causes of arrhythmia in HF and these are comprehensively reviewed elsewhere (Pogwizd & Bers, 2004; Scoote & Williams, 2004; Tomaselli & Zipes, 2004).

#### 4.1.1 Changes in protein expression

HF is mostly a progressive disease, which involves remodelling of the whole organ and of the cardiomyocyte and many of the changes in myocyte function may result from altered patterns of gene expression (Swynghedauw, 2006). Both PKA (Pare et al., 2005; Bauman et al., 2007) and CaMKII (Maier & Bers, 2002) phosphorylate components of gene transcription pathways and thus have dual roles in ECC and excitation–transcription coupling. The latter involves regulation of gene expression in response to raised Ca^2+^ (Bare et al., 2005; Wu & Bers, 2006). In this context LCC activity is linked to regulation of K^+^ channel expression (Richard et al., 2006). Ca^2+^/CM provides a link to calcineurin via the NFAT transcription factor (Jones et al., 2003), which is for example activated during the progression of pathological hypertrophy (Wilkins et al., 2004).

Changes in expression of most of the proteins associated with the ECC cycle have been reported in HF in both animal models and human heart. Generally expression of SERCA is depressed (Williams et al., 1994; Kiss et al., 1995; Zarain-Herzberg et al., 1996) and the extent may mirror the severity of the symptoms seen in human failing and non-failing hearts (Hasenfuss et al., 1994) and be time dependent (Sallinen et al., 2007). Conversely in human heart NCX is increased (Hasenfuss et al., 1994; Reinecke et al., 1996; O'Rourke et al., 1999; Schwinger et al., 1999b; Pogwizd, 2000; Litwin & Zhang, 2002). This combination of increased NCX and decreased SERCA expression favours increased cellular extrusion and decreased store Ca^2+^, limiting cytosolic Ca^2+^ available for contractile protein deinhibition. Phosphatase inhibitor protein (P-1), which inhibits PP1 activity, is decreased in human HF (El-Armouche et al., 2004) and PP1 levels were increased in a rat model of HF (Huang et al., 1999). Thus phosphatase activity would be increased and PLB in particular would be dephosphorylated limiting SERCA activity and uptake into Ca^2+^ stores. RyR2 expression has been reported to be decreased in HF by 25% in rabbit (Ai et al., 2005) or ∼30% in canine (Song et al., 2005) models of aortic insufficiency, 48% in a canine model of chronic HF by pacing (Kubalova et al., 2005) and 35% in human heart (Go et al., 1995).

RyR2 is regulated by a number of accessory proteins many of which are reported to co-immunoprecipitate as part of a macromolecular complex (Bers, 2004), which includes a number of proteins — phosphodiesterase 4D (PDE4D3) (Lehnart et al., 2005b), FKBP12.6 (Collins, 1991; Jayaraman et al., 1992), PKA, PP1 and PP2A (Marx et al., 2001b) and CaMKII (Currie & Smith, 1999; Wu et al., 2001; Zhang et al., 2003b; Wehrens et al., 2004b), Fig. 3. FKBP12.6, PP2A and PPI are reported to be lost from the complex in HF in a paced dog model (Marx et al., 2000; Yano et al., 2000) and in a rabbit model of aortic constriction (Ai et al., 2005) and in human HF (Marx et al., 2000). Decrease in expression levels or binding affinity for RyR2 may both be contributory factors and the evidence for the individual changes are discussed in the appropriate sections below.

#### 4.1.2 Molecular mechanisms of ryanodine receptor type-2 dysfunction

The cause of RyR2 dysfunction is highly controversial and may involve a number of potential mechanisms. The hyperphosphorylation hypothesis (Marx et al., 2000) proposes that the loss of phosphatases is responsible for the hyperphosphorylation of RyR2 and that this caused the loss of the modulator protein FKBP12.6 from the RyR2 complex (Marx et al., 2000; Yano et al., 2000). This would cause instabilities in RyR2 channel gating leading to increased diastolic leak from RyR2 setting up the conditions to generate DADs. This hypothesis, however, it is not without its detractors but it has provided both the impetus and focus for a lively debate about the mechanisms underlying RyR dysfunction. Another hypothesis concerns interacting domains of the RyR2 molecule that ‘unzip’ as part of the molecular rearrangement regulating channel gating between the open and closed conductive states — interactions which may be susceptible to oxidation (Oda et al., 2005; Yano et al., 2005). Increased sensitivity of RyR2 to luminal Ca^2+^ has also been reported in HF (Kubalova et al., 2005).

##### 4.1.2.1 Ryanodine receptor type-2 hyperphosphorylation

The development of HF is associated with increased levels of circulating catecholamines and persistant β-adrenergic stimulation which, as a protective compensatory mechanism, leads to decreased receptor expression and cAMP production by adenyl cyclase and hence decreased PKA activation (Bristow et al., 1982). In this regard, the deleterious effects of persistent PKA activation have been illustrated by constitutive expression of the active subunit of PKA in transgenic mice, which resulted in cardiomyopathy, decreased contractility, arrhythmia and susceptibility to SCD (Antos et al., 2001). Conversely, symptoms of HF, including restored SR function and reduced Ca^2+^ leak, can be ameliorated by pretreating paced dogs with β-blockers (Reiken et al., 2001; Doi et al., 2002) or the AT1 receptor antagonist — valsartan, which inhibits noradrenaline release from the synaptic pool (Tokuhisa et al., 2006).

Many proteins in ECC are co-ordinately upregulated by PKA phosphorylation during the normal ‘flight or fight’ response resulting in faster Ca^2+^ cycling. This upregulation is ultimately balanced by dephosphorylation by phosphatases (Bers, 2002), Fig. 3. In HF PP1 protein levels are reported to be increased (Neumann et al., 1997; Huang et al., 1999) and expression of its inhibitor PI-1 decreased (El-Armouche et al., 2004), which should predict hypophosphorylation, as is reported for PLB (Huang et al., 1999; Dash et al., 2001) with residues T16 and T17 showing reduced phosphorylation (Schwinger et al., 1998; Huang et al., 1999). This may be one of the causative factors for the reduced SR Ca^2+^ store in HF as SERCA activity is repressed by dephosphorylated PLB (MacLennan & Kranias, 2003). Animal models of phosphatase overexpression can cause HF by producing hypophosphorylation of key ECC proteins (Carr et al., 2002; Gergs et al., 2004; Kirchhefer et al., 2005). Conversely overexpression of PI-1/2, the phosphatase inhibitory protein can rescue HF and up-regulate SERCA activity (via PLB) and raise SR store Ca^2+^ (Pathak et al., 2005; Yamada et al., 2006). Perversely, RyR2 is reportedly hyperphosphorylated in a number of studies (Marx et al., 2000b; Yano et al., 2000; Ai et al., 2005; Song et al., 2005) but unchanged in others (Jiang et al., 2002b; Xiao et al., 2005). Thus disruption of the fidelity of ECC in HF can be viewed as the loss of co-ordinated regulation by phosphorylation (Sipido & Eisner, 2005).

###### 4.1.2.1.1 Why is ryanodine receptor type-2 hyperphosphorylated?

The crux of the RyR hyperphosphorylation hypothesis is its microdomain complex (Bers, 2004), from which key proteins can be lost in isolation from the general cellular milieu. These include the phosphatases PP1 and PP2A in the paced dog model (Marx et al., 2000; Yano et al., 2000), human failing heart (Marx et al., 2000) and in a rabbit model of aortic constriction (Ai et al., 2005). In addition, PDE4D3 (which metabolises cAMP) is reduced in human HF and PDE4D3-deficient mice have been shown to develop cardiomyopathy and exercise-induced arrhythmias (Lehnart et al., 2005b). It is proposed that, in the vicinity of the RyR2 complex/microdomain, cAMP levels would remain high because PDE4D3 is lost, thus activating PKA phosphorylation of RyR2. Phosphorylation would persist because the phosphatases PP1 and PP2A have also been lost from the complex (Marks, 2003).

###### 4.1.2.1.2 What is hyperphosphorylation?

There are several RyR2 phosphorylation sites and there is no consensus view on which are fully phosphorylated in HF or those that are the most important. The S2809 residue has been shown experimentally to be hyperphosphorylated in HF in a paced dog model and in myocardium from human transplant (Marx et al., 2000). The importance of the S2809 residue was further emphasised using a knock-in mouse model with an S2809A mutation site — a residue which could not be PKA phosphorylated, and these mice did not develop such severe symptoms of HF, following myocardial infarction, as WT mice (Wehrens et al., 2006). These same S2809A mice were also protected when given an antagonist to inhibit PDE4D3 (hence raise cAMP) and challenged with isoprenaline, a regime, which caused hyperphosphorylated RyR2 and fatal arrhythmia in WT (Lehnart et al., 2005b). In contrast, using a paced dog model it has been shown that S2809 phosphorylation was unchanged in HF (Jiang et al., 2002b; Xiao et al., 2005) and a different residue — S2030, showed PKA-dependent phosphorylation (Xiao et al., 2005).

A number of studies have explored the role of CaMKII in hypertrophy and HF in both human (Hoch et al., 1999) and animal studies (Currie & Smith, 1999; Hagemann et al., 2001; Ai et al., 2005). CaMKII, like PKA, is upregulated in hypertrophy and HF and shown to phosphorylate LCC, PLB on T17 and RyR2 (Zhang & Brown, 2004). Three-fold over expression of CaMKII in a transgenic mouse model resulted in HF at three months and the isolated cardiomyocytes showed a 50% reduction in SR store Ca^2+^, with reduced SERCA and increased NCX expression together with an increase in spontaneous Ca^2+^ sparks suggesting diastolic leak from RyR2 (Maier et al., 2003). Another study, in a rabbit model of aortic constriction, measured increases of 63% in total CaMKII protein and 43% in autophosphorylated CaMKII with an increase in phosphorylation of PLB at T17 (and decreased phosphorylation of S16) and both S2809 and S2815 of RyR2 were phosphorylated (Ai et al., 2005). In the same study, by using either a PKA (H-89) or CaMKII (KN-93) inhibitor, it was shown that KN-93 could decrease diastolic leak from SR and increase the Ca^2+^ transient whereas inhibiting PKA activity had no effect. This suggested that the major protagonist of RyR hyperphosphorylation in HF was CaMKII and not PKA (Ai et al., 2005). In support, it has been shown that CaMKII inhibition by inhibitory peptides suppressed transient inward currents, which originated from NCX reversal and which were associated with DADs (Wu et al., 1999).

###### 4.1.2.1.3 How does hyperphosphorylation modulate ryanodine receptor type-2?

One of the problems with answering this question is the lack of consensus in experimental results and indeed any real mechanistic understanding of how phosphorylation normally alters RyR channel gating. This has been outlined in Section 2.4. Phosphorylation by CaMKII may regulate RyR2 differently from PKA as, apart from S2809, they have different phosphorylation sites (Rodriguez et al., 2003). It is proposed that hyperphosphorylation of RyR2 perpetrates the loss of the modulatory protein FKBP12.6 (Marx et al., 2000) and it is this which results in RyR2 dysfunction.

##### 4.1.2.2 FKBP12.6 loss and ryanodine receptor type-2 dysfunction

FKBP12.6 is a potent regulator of RyR2 function and the loss (by addition of FK506) of FKBP12/12.6 from normal channels results in RyR dysfunction including increased *P*_o_ and subconductance states in single channel studies (Brillantes et al., 1994; Chen et al., 1994; Ma et al., 1995) and increase in Ca^2+^ spark frequency in cardiomyocytes (Gomez et al., 2004; Loughrey et al., 2004). The relationship between FKBP12.6 loss and phosphorylation causing arrhythmia has been explored in FKBP12.6 ^-/-^ and ^+/-^ cross bred mice (Xin et al., 2002; Lehnart et al., 2006). Disconcertingly the models showed different characteristics. In one, no arrhythmia was seen at rest and isoprenaline injection was necessary to produce arrythmogenic beats (Lehnart et al., 2006). In the other CICR gain was increased at rest — manifest as an increase in the amplitude and duration of spontaneous Ca^2+^ sparks (Xin et al., 2002). The reason for this difference was not clear, but both models illustrate that there is compensation for the loss of FKBP12.6 which is not lethal.

It has been proposed that in HF hyperphosphoryation by PKA of S2809 specifically causes the loss of the RyR2 modulatory protein FKBP12.6 (Yano et al., 2000) whereas CaMKII (Wehrens et al., 2004b) or PKC did not cause a similar dissociation (Marx et al., 2000; Wehrens et al., 2004b), although CaMKII is reported to phosphorylate S2809 (Witcher et al., 1991; Marx et al., 2000; Rodriguez et al., 2003). A decrease in the ratio of [^3^H]FK506 to [^3^H]ryanodine was measured in microsomal fractions prepared from a 4 week paced dog model of HF (Yano et al., 2000). A fluorescent probe technique, which measured conformational change in RyR2 on the addition of FK506, showed little change in fluorescence in the paced dogs implying that the FKBP12.6 had already been lost (Yano et al., 2000). Using the same paced dog model a second group also showed a loss of FKBP12.6 together with decreased levels of PP1 and PP2A in a RyR2 complex that was solubilised from microsomes (Marx et al., 2000). The loss of FKBP12.6 was attributed to the hyperphosphorylated state of RyR2 since in one study, phosphorylation of RyR2 from control dogs was shown to cause the loss of FKBP12.6, specifically by PKA at S2809 and furthermore, these effects could be reversed by fitting the paced dogs with a left ventricular assist device (LVAD) which aided recovery of contractile function (Marx et al., 2000).

One study showed a decrease of 66% in FKBP12.6 associated with SR (paced dog) (Yano et al., 2000) but another has shown no change in the paced dog model or human HF (Jiang et al., 2002b). Other studies, measuring the FKBP12.6 that can be immunoprecipitated with the solubilised RyR2 complex, demonstrated a decrease in the FKBP12.6/RyR2 ratio of 50% (paced dog) or 65% (human HF) (Marx et al., 2000) or 38% (rabbit aortic insufficiency) (Ai et al., 2005).

###### 4.1.2.2.1 What might cause FKBP12.6 loss?

For RyR2 to lose FKBP12.6 from its binding site the binding affinity must either change or the concentration of FKBP12.6 must fall and there is evidence for both.

It has been proposed that the addition of the negatively charged phosphate onto RyR2 S2809 by PKA results in a charge repulsion preventing FKBP12.6 binding (Wehrens et al., 2006). Removing the negatively charged D37 residue of FKBP 12.6 — a part of its binding domain to RyR2 by substitution with V or S increased FKBP12.6 binding to PKA phosphorylated SR vesicles or the S2808D RyR2 mutant expressed in HEK293 cells (Wehrens et al., 2003). Single channel studies showed that binding the D37V mutant could decrease the *P*_o_ of S2808D RyR2 and ‘stabilise’ channel activity (Wehrens et al., 2006). However, it is unlikely that there is a direct interaction between the proposed phosphorylation site(s) (Jones et al., 2007; Meng et al., 2007) and FKBP12.6 (Wagenknecht et al., 1997) as these map on the topology models to different regions of the RyR structure — Fig. 4. This would give credence to the view that phosphorylation invokes an internal molecular rearrangement of RyR which alters the affinity for FKBP12.6 (Jones et al., 2006). Other studies have also examined the phosphorylation status of S2808 and FKBP12.6 binding using HEK293 cells and both concluded that there was no relationship. Expression of mutant RyR2 S2808D or S2808A and its RyR1 homologue S2843D or S2843A, showed no change in the binding to FKBP12.6/12, of the mutated RyR channels and no differences the Ca^2+^ dependence of single channel activity, although the latter experiment did not include the effect of FKBP12.6/12 addition (Stange et al., 2003). Similar experiments, and in addition, pull-down assays from microsomes of canine cardiac muscle with either GST-FKBP12.6 or antibody to RyR2, showed an unchanged association of FKBP12.6 to RyR2 whether or not the latter was phosphorylated (Xiao et al., 2004).

Using Biacore technology, to measure the equilibrium binding kinetics of interaction between RyR1 and FKBP12 it was shown that affinity of solubilised RyR1 for FKBP12 is lower for the open channel than the closed (Jones et al., 2005) and this is also true for RyR2 (unpublished observations). The effect of PKA phosphorylation was to shift the affinity of the closed channel to the lower affinity of the open channel, nonetheless, the affinity for the open state was still high (Jones et al., 2005). It was concluded that the shift in affinity was part of the normal ‘flight or fight’ response mechanism. The equilibrium binding data was interpreted as demonstrating a change in conformation of RyR when phosphorylated — possibly a molecular movement to a state which would favour the open/closed transition (Jones et al., 2006). This is consistent with conclusions from single channel studies (Valdivia et al., 1995) and in myocytes from PLB-DM mice (Ginsburg & Bers, 2004), that RyR2 must open and close more rapidly to facilitate upregulation of ECC by phosphorylation. The domain zipping hypothesis also predicts a molecular movement within the RyR2 molecule (Oda et al., 2005), see Section 4.1.3. A decrease in affinity of FKBP12.6 to the open or phosphorylated channel could translate into a loss of FKBP12.6 during the isolation of either the RyR2 complex or SR vesicles, since the rate of dissociation would be greater once RyR2 was no longer exposed to free FKBP12.6, within its microdomain environment.

FKBP12.6 distribution has been difficult to determine at the cellular level as cardiac muscle contains high levels of the FKBP12 isoform as well as the cardiospecific FKBP12.6 (Timerman et al., 1996) and no distinguishing antibodies have been available. Measuring mRNA FKBP12.6 and FKBP12 levels showed 49% and 20% decreases respectively in HF (rabbit banded aorta) which corresponded to a 38% decrease in the FKBP12.6/RyR2 ratio (Ai et al., 2005).

There are many unresolved issues surrounding the relationship between FKBP12.6 and RyR2. In particular, it is not at all clear in those studies in which FKBP12.6 is lost in HF, whether this is due to reduced expression or decrease in affinity.

#### 4.1.3 Oxidation and domain unzipping

Evidence for the contribution of oxidative stress to the development of HF is considered by some to be inconclusive (Mak & Newton, 2001; Giordano, 2005). However, others propose that there is good evidence that oxidative stress is involved in many of the pathological remodelling processes that ultimately lead to HF (Sawyer et al., 2002). Several of the protein components of ECC including RyR are modified by oxidation (Choudhary & Dudley, 2002; Pessah et al., 2002). RyR contains a number of active cysteine residues which are susceptible to modulation by the redox state of the channel (Xu et al., 1998; Sun et al., 2001). Oxidation of RyR increases ryanodine binding (Xia et al., 2000) and increases *P*_o_ in bilayer studies (Marengo et al., 1998). For reviews see (Zima & Blatter, 2006; Zissimopoulos & Lai, 2006). Using a 4 week paced dog model the synthetic antioxidant edaravone was able to improve the haemodynamic properties and contractile function of myocytes, improve SR function by restoring SERCA protein levels and reverse hyperphosphorylation of RyR restoring FKBP12.6 binding (Yano et al., 2005). *In vitro* studies, using pull down assays has shown that oxidation of RyR2 can reduce binding to recombinant FKBP12.6 (Zissimopoulos & Lai, 2006). Exposure to reactive oxygen species, produced during the development of HF, could cause domain unzipping and edaravone could prevent this (Choudhary & Dudley, 2002).

The DPc10 peptide was used to target a fluorescent indicator into the zipper domain where it was cross-linked to RyR2 protein (Yamamoto & Ikemoto, 2002). A fluorescence quencher attached to BSA could only access the domain when unzipped and was used to measure state of this domain in SR from both normal and HF (paced dog). It was shown that this domain could be unzipped in normal SR by the oxidant SIN-1 (Yano et al., 2005) but was already unzipped in HF (Oda et al., 2005). In SR from control dogs, application of cAMP to phosphorylate RyR could also unzip the domain and this was associated with loss of FKBP12.6, implying that phosphorylation could cause domain unzipping but unzipping by DPc10 alone did not dissociate FKBP12.6 (Oda et al., 2005).

#### 4.1.4 Ryanodine receptor type-2 sensitivity to luminal Ca^2+^

Some studies have reported increased numbers of Ca^2+^ sparks in myocytes isolated from failing hearts despite reduced store Ca^2+^ and RyR2 expression (Kubalova et al., 2005; Song et al., 2005). One study, using a chronic HF model of paced right ventricle of canine heart (13–24 months), included single channel measurements of RyR2 function and these demonstrated increased sensitivity to luminal Ca^2+^ (Kubalova et al., 2005). This might provide the explanation for increased Ca^2+^ spark activity despite a decreased store Ca^2+^ and it has been proposed that PKA phosphorylation increases the sensitivity of RyR2 to luminal Ca^2+^ (Xiao et al., 2007a).

#### 4.1.5 Delayed after-depolarisations in heart failure

There is sufficient evidence, particularly from experimental models to suggest that RyR2 function in HF is perturbed and that diastolic leak is a feature of HF even if there is no agreement on the precise mechanism of dysfunction. These changes in RyR2 function in HF take place against a greatly modified cellular landscape, which could also contribute to the propensity for arrhythmia. The close spatial relationship between T-tubule and junctional SR — the dyadic cleft, which is so important to the electrophysiological homeostasis of the heart beat can become disrupted in HF (Balijepalli et al., 2003; Izu et al., 2006; Song et al., 2006; Bito et al., 2008; Chen-Izu et al., 2007). Co-localisation between RyR2 and LCC is disturbed as T-tubules are lost from the Z-line positions leaving junctional RyR2 ‘orphaned’ (Song et al., 2006). This will have serious repercussions for the critical interplay between RyR2 activation and retrograde Ca^2+^ dependent inactivation of LCC (from RyR2 Ca^2+^ release), as seen in a detubulated myocyte model (Brette et al., 2004). The ratio of RyR2 to LCC was altered in one study (Milnes & MacLeod, 2001) and a functional uncoupling was proposed in another, where the ratio remained the same (Gomez et al., 1997). The interaction between the other ion channels and exchangers which contribute to the AP (NCX, K^+^ and Na^+^ channels and the Na^+^/K^+^ ATPase) that interact functionally through the Ca^2+^, K^+^ and Na^+^ ionic changes in the dyadic cleft will also be compromised by such structural changes.

AP duration is increased in human HF (Davia et al., 2001; Kogler et al., 2003; Stagg et al., 2004) and animal models (Huang et al., 2000; Milnes & MacLeod, 2001; Perrier et al., 2004). Electrical remodelling and down regulation of K^+^ channels occurs early in the development of hypertrophy (Huang et al., 2000; Perrier et al., 2004). A down-regulation of the transient outward current *I*_to_ and a decrease in mRNA for the K^+^ channel K_v4.2_ has been reported that may involve a central role for LCC in regulation of gene expression (Perrier et al., 2004). However, LCC protein expression itself, in most studies, is unchanged in HF (Richard et al., 2006). Many studies show upregulation of NCX expression two or three fold (Hasenfuss et al., 1994; Reinecke et al., 1996; O'Rourke et al., 1999; Schwinger et al., 1999b; Pogwizd, 2000; Litwin & Zhang, 2002). The inward rectifying K^+^ current (*I*_KI_) normally stabilises the resting *E*_m_ but its activity is reduced by Ca^2+^ (Fauconnier et al., 2005). The increased diastolic leak of Ca^2+^ in HF may contribute to destabilisation of *E*_m_ and together with the increase in NCX could lower the threshold for DADs, so that even a modest diastolic leak from RyR2 may result in an increased propensity to trigger a fatal arrhythmia (Pogwizd & Bers, 2002; Scoote & Williams, 2004; Fauconnier et al., 2005; Sipido & Eisner, 2005).

### 4.2 Catecholaminergic polymorphic ventricular tachycardia

#### 4.2.1 Clinical presentation and genotype

There are a number of inherited arrhythmias affecting ion channel function (Lehnart et al., 2007). CPVT is a rare, inheritable autosomal dominant condition (Mohamed et al., 2007), which was described before genotyping became available, in patients with family members who developed tachyarrhythmic episodes associated with emotional stress or exercise or had died suddenly (Priori et al., 2002) often in childhood or adolescence (Coumel et al., 1978). It is associated with a 30–35% death rate by the age of 30 (Priori et al., 2002). The first mutations in the RyR2 gene found to be associated with CPVT1 were described in 2001 (Priori et al., 2001), although CPVT1 had been mapped to the chromosome 1 1q42–q43 locus in 1999 (Swan et al., 1999). 69 point mutations were identified in the RyR2 gene in 253 (out of 915) patients screened for CPVT1 and this number is growing as more patients are genotyped — a current list of mutations is available on the ESC ‘Gene connection for the heart’ data base (http://www.fsm.it/cardmoc). Some of these RyR2 mutations have been identified in patient groups screened for Long QT syndrome (Tester et al., 2005b), arrythmogenic right ventricular cardiomyopathy type 2 (ARVD2), in which there is histological evidence of fibrofatty streaks in the right ventricle and the tachycardia exhibits polymorphic characteristics (Tiso et al., 2001; Moric-Janiszewska & Markiewicz-Loskot, 2007) and unexplained drowning on post mortem (Tester et al., 2005a). In addition, a few patients have been identified with CPVT symptoms but have mutations in the RyR2 luminal modulator protein calsequestrin, *Casq2* gene (CPVT2) which is linked to chromosome1p13–p21 but these patients have an autosomal recessive phenotype (Lahat et al., 2001b; Postma et al., 2005; Terentyev et al., 2006).

The arrhythmia displayed by CPVT patients is characterised in the ECG by a distinctive bidirectional ventricular tachycardia or polymorphic ventricular premature beats that occur with stress or exercise but which are not associated with a prolonged QT interval (Leenhardt et al., 1995; Priori et al., 2002; Postma et al., 2005), although two studies show that the resting ECG may show lower QT intervals (Tester et al., 2005b). A slower heart rate may also be typical (Leenhardt et al., 1995) and was observed when compared with both non carriers within a family and unrelated controls (Sumitomo et al., 2003; Postma et al., 2005).

CPVT is a relatively rare disease estimated to account for ∼ 6000 unexplained SCDs (with no underlying cardiac pathology) in Europe and the USA annually (George et al., 2006). The numbers of patients with a known genotype are currently small. Nonetheless patterns are emerging as to the criteria for diagnosis (Napolitano & Priori, 2002; Sumitomo et al., 2003; Napolitano et al., 2005; Postma et al., 2005) and treatment of CPVT (Napolitano et al., 2006), aetiopathology (Chugh et al., 2004; George et al., 2006) and molecular genetics (Napolitano & Priori, 2002). This section will concentrate on the molecular mechanisms, which underlie the phenotype.

#### 4.2.2 Catecholaminergic polymorphic ventricular tachycardia-1 mutational clusters

The RyR2 point mutations are traditionally grouped into three regions of the RyR molecule N-terminus, central domain and C-terminus clusters which are largely homologous to similar groups of point mutations in the RyR1 gene (see Dirksen & Avila, 2002, for a list of RyR1 mutations). There is structural/functional evidence to split the C terminus mutational cluster in RyR2 into two regions as discussed later (George et al., 2006). See Fig. 4 for a diagrammatic representation of these mutational clusters spaced on the RyR2 sequence.

The first RyR point mutation was detected in the skeletal muscle RyR1 isoform a decade earlier than the RyR2 counterparts and this was the R615C mutation causing porcine stress syndrome (Fujii et al., 1991). Many mutations of RyR1 have now been linked to the human counterparts of this condition — malignant hyperthermia (MH) and central core disease (CCD) (MacLennan et al., 1990; Chen et al., 1993; Quane et al., 1993), which can have overlapping phenotypes (Frank et al., 1980; Dirksen & Avila, 2002). MH manifests itself as an extreme reaction to volatile anaesthetics, such as halothane and muscle relaxants such as succinylcholine, resulting in rapid onset of muscle rigidity, unstable and increasing blood pressure and high temperature, and CCD with muscle weakness and characteristic remodelling of skeletal muscle sarcomeres seen in histological sections (Dirksen & Avila, 2002).

Using SR from human biopsy of a carrier of the G2434R mutation, ryanodine binding studies showed the mutant channel to have an increased sensitivity to caffeine and 4-chloro-*m*-creosol and a reduced sensitivity to inhibiting concentrations of Ca^2+^ and calmodulin (Richter et al., 1997). The testing of muscle strips with caffeine and halothane was developed as the *in vitro* contracture test (IVCT) a diagnostic test for MH although genetic screening is now also available (Urwyler et al., 2001). Most experimental models of MH and CCD involve the transient expression of mutant RyR1 in HEK293 or dyspedic muscle cells (Tong et al., 1999; Dirksen & Avila, 2004) and much of the data is consistent with the human study — namely that SR Ca^2+^ leak of varying severity is associated with the MH and the MH plus CCD phenotype, although uncoupling of activation of RyR from allosteric activation by LCC and a resultant reduction of Ca^2+^ release is a feature of some CCD mutations (Avila et al., 2001). To explain the relative severity of mutations associated with MH, MH + CCD and CCD a schematic, based on the characteristics of a number of point mutations expressed in dyspedic myotubes has been proposed (Dirksen & Avila, 2004). The MH mutations showed an increase in spontaneous Ca^2+^ release events and an increased sensitivity to activation (voltage sensitive Ca^2+^ release). Those associated with the MH plus CCD, in addition, showed underlying SR Ca^2+^ store depletion and CCD mutations had SR Ca^2+^ store depletion but also a decreased response to activation — EC uncoupling (Dirksen & Avila, 2004). The mutations and their severity did not strictly segregate within the three defined clusters although mutations associated with CCD (33 out of 44) tended to be in the C-terminus region (Lynch et al., 1999; Monnier et al., 2001), however the CCD phenotype was also seen in a few mutations in the N-terminus and central domain clusters (Quane et al., 1993; Zhang et al., 1993; Dirksen & Avila, 2004; Xu et al., 2006) (for reviews on MH and CDD refer to Dirksen & Avila (2002), Dulhunty et al. (2006), and Robinson et al. (2006)).

The observations for RyR1 can be generally translated to RyR2 allowing for the fact that CICR is the only mechanism of activation of RyR2 and not a direct allosteric interaction with LCC. Only a few of the RyR2 mutations identified in patients have been characterised. However those which have mostly exhibit gain of function features which can broadly be defined as an increased open probability in response to activation by caffeine, 4-chloro-m-creosol or phosphorylation compared to WT.

Two models of knock-in mice have been created carrying the CVPT1 mutations R4496C (human mutation R4497C) (Cerrone et al., 2005; Liu et al., 2006) and the ARVD2 mutation R176Q (Kannankeril et al., 2006). Both models showed normal function at rest but arrhythmic episodes were provoked by isoproterenol or caffeine and epinephrine administration. Isolated cardiomyocytes from the R176Q mice showed an increased numbers of spontaneous Ca^2+^ oscillations even at rest and a mixture of aberrant Ca^2+^ transients, when stimulated with isoproterenol, consistent with the generation of DADs but SR store Ca^2+^ was unaltered (Kannankeril et al., 2006). The R4496C mutation also exhibited DADs in paced myocytes, which, in the presence of isoproterenol, increased and were sustained after cessation of pacing (the WT also showed some DAD activity with isoproterenol but only when paced) (Liu et al., 2006). The R176Q mutation showed no histological evidence typical of human ARVD2 changes in the right ventricle but there was evidence of cardiomyopathy, in 1 year old mice, in the form of decreased end-diastolic volume of the right and left ventricles and higher end-diastolic pressure in the left ventricle (Kannankeril et al., 2006). Epicardial and endocardial optical mapping suggested that the arrhythmias originated from His-Pukinje networks in the right ventricle and/or the left ventricle in the R4496C mouse and in addition single isolated Purkinje cells demonstrated DADs even without stimulation by isoproterenol (Cerrone et al., 2007). This work also addressed various patterns of arrhythmia seen on ECG and the focal origin within the conduction system provided an explanation for the transitions between biventricular, polymorphic and ventricular fibrillation characteristic of CPVT (Cerrone et al., 2007).

The CPVT2 CSQ D307H knock-in mouse model had structurally normal hearts and ventricular function (Dirksen et al., 2007). There were no changes in the expression of other ECC proteins, although the levels of mutant CSQ varied with individual transgenic lines showing up to 6 fold expression. Myocytes displayed an increase in spark frequency and a decrease in spark amplitude and in the presence of caffeine, the addition of isoproterenol invoked spontaneous Ca^2+^ transients. ECG registered complex ventricular arrhythmias when isoproterenol (with or without caffeine) was administered (Dirksen et al., 2007).

Thus, the knock-in mouse models largely confirm the human phenotypes that are associated with RyR2 mutant dysfunction, and support the view that a trigger, such as exercise or emotional stress, (isoprenaline or caffeine plus epinephrine in mouse models) could evoke DADs (Nakajima et al., 1997).

#### 4.2.3 Molecular mechanisms of ryanodine receptor dysfunction

The sub-cellular manifestation of increased adrenergic drive is PKA phosphorylation of key ECC proteins (including RyR2), which cause changes in the RyR2 microdomain environment as outlined above in Section 2.4. The finding that CSQ mutants also cause the CPVT2 phenotype puts the focus on aberrant Ca^2+^ signalling, translated through RyR2 modulation and possibly manifest through SR store Ca^2+^ concentrations. Simulations of CPVT1 and CPVT2 have tested some of these proposed mechanisms, which might underlie abnormal Ca^2+^ homeostasis. These models were able to predict DADs (in response to β-adrenergic stimulation) induced by impaired luminal Ca^2+^ sensing in CPVT2 (Faber & Rudy, 2007; Iyer et al., 2007) and also consistent with store-overload-induced Ca^2+^ release (SOICR) in CPVT1 (Iyer et al., 2007). Impaired coupled gating, due to reduced FKBP12.6 binding to RyR2, could also produce simulated DADs (Iyer et al., 2007). Thus some proposed mechanisms for dysfunction are emerging and the molecular characterisation of the individual CPVT mutations are discussed within the context of these.

##### 4.2.3.1 Ryanodine receptor interdomain interactions

Several observations have led to the hypothesis that the clusters of RyR1/2 point mutations may be coincident with one or more domain interactions. In CPVT, mutations fall into the N-terminus or central domain clusters, which make up the proposed interacting zipper domains (Yamamoto & Ikemoto, 2002) or into the C-terminus domain, which is divided into the ‘I-domain' and the transmembrane region (George et al., 2004). It is proposed that a mutation in any of the domains would weaken the interaction with its partner thus causing instability and loss of fidelity of channel gating.

###### 4.2.3.1.1 Zipper domain

The synthetic peptide DP4 (2442–2477 of RyR1) was able to disrupt the zipper domain interaction, whereas DP4 with the R2458C MH mutation had no effect (Yamamoto et al., 2000). When introduced into permeabilised frog skeletal muscle fibres peptide DP4 increased Ca^2+^ spark frequency but DP4 (R2458C) had no such effect (Shtifman et al., 2002). The DP4 peptide was also able to increase ryanodine binding in SR vesicles but DP4 peptides containing the mutations R2452W, I2453T, R2454C, R2454W, R2458C or R2458H all showed greatly reduced activation (Bannister et al., 2006). NMR analysis of DP4 showed it to comprise two helical regions with the cluster of MH mutations (R2452W, I2453T, R2454C, R2458C or R2458H located at the C-terminus of the first helix or in the loop connecting the two helices (Bannister et al., 2006). It was shown, using a similar peptide DPc10 (G2460–P2495), for RyR2 studies (Yamamoto & Ikemoto, 2002) containing the CPVT mutation R2474S (Priori et al., 2001) that the WT peptide increased the sensitivity to activating Ca^2+^ and increased ryanodine binding but DPc10M (R2474S) had no effect (Yamamoto & Ikemoto, 2002). The implication from these studies was that a RyR point mutation, introduced to a modulatory peptide, could render it incapable of normal domain interaction.

###### 4.2.3.1.2 I-domain

The C-terminus cluster of CPVT mutations can be sub-divided into two regions 3534–4610 (the I-domain (George et al., 2004)) and 4497–4959 (the transmembrane/pore region (Williams et al., 2001; Du et al., 2002)). Using a fusion protein complementation approach and FRET analysis it was demonstrated that an N terminus − 4610 construct containing the I-domain and labelled with DsRed could regulate the RyR2 C-terminus, tagged with GFP (the latter expressed in CHO cells in an induceable system) and could restore caffeine sensitivity. A CPVT mutation either in the N-terminus construct (S2246L) or in the C-terminus (N4104K or R4497C) showed similar basal regulation of Ca^2+^ when expressed in CHO cells. However, when challenged with caffeine all of the mutant constructs showed an increased sensitivity (George et al., 2006). Noise analysis, of the FRET signal, distinguished the two C-terminus mutations from that in the N-terminus construct and was attributed to instabilities in the I-domain regulation of the C-terminus. These effects were also observed when the noise analysis was extended to the mutant channels expressed in HL-1 cells (cardiac phenotype) indicating that the instabilities were attributable to the RyR2 protein itself and not to the absence (CHO cells) of RyR2 regulatory proteins (George et al., 2006).

The I-domain/C-terminus interaction was explored in RyR1 using the DP15 (4821–4841) peptide (Hamada et al., 2007) corresponding to the putative cytoplasmic loop connecting transmembrane regions 6 and 7 (Du model (Du et al., 2002)). Activation of RyR1 by DP15 was lost when the L4823P or L4837V MH causing mutations were introduced (shown in ryanodine binding experiments). The corresponding I-domain interaction site was a 92 kDa fragment of RyR1, which was recognised by an anti-RyR1 site-specific Ab to 4114–4142. In analogy to the effect of DPc10 on the ‘zipper domain’ interaction it was proposed that the I-domain/C-terminus interaction stabilises the closed channel and this is compromised by point mutations (Hamada et al., 2007).

##### 4.2.3.2 Regulation by FKBP12.6

It has been proposed that a CPVT mutation alters FKBP12.6 binding characteristics. CPVT mutations (S2246L, R2474S or R4497C (Wehrens et al., 2003) or P2328S, Q4201R or V4653F (Lehnart et al., 2004)) were co-transfected with FKBP12.6 into HEK293 cells. All mutants exhibited normal single channel properties with 150 nM Ca^2+^ in the *cis* chamber but PKA phosphorylation increased the *P*_o_ of all channels and some sub-conductance states were seen. The mutants were also more sensitive to activating Ca^2+^ concentrations (S2246L, R2474S or R4497C (Wehrens et al., 2003)) and P2328S (Lehnart et al., 2004) and inhibition by Mg^2+^ was reduced (P2328S, Q4201R or V4653F (Lehnart et al., 2004)). The mutant channels all showed reduced (2 fold) affinity for FKBP12.6, which did not alter their single channel activity at rest, but was manifest only when the channels were phosphorylated (Wehrens et al., 2003; Lehnart et al., 2004). The ‘sticky’ FKBP12.6 D37S mutant could bind to both WT and the R2474S mutant even when phosphorylated and reduce *P*_o_ to WT non-phosphorylated levels suggesting that deficient FKBP12.6 binding was part of the CPVT mechanism (Wehrens et al., 2003).

##### 4.2.3.3 Catecholaminergic polymorphic ventricular tachycardia-1 and regulation by luminal Ca^2+^

The SOICR (store overload induced Ca^2+^ release) hypothesis postulates that the aberrant feature of CPVT mutations is an enhanced sensitivity to luminal Ca^2+^ concentration (Jiang et al., 2004, 2005). A number of mutations were examined by expression in HEK293 cells — C-terminus mutations N4104K, Q4201R, R4496C, I4867M and N4895D, central domain mutants S2246L and R2474S and N terminus mutants R176Q/T2504M and L433P (Jiang et al., 2004, 2005). All mutations increase the frequency of spontaneous Ca^2+^ oscillations seen in both HEK293 cells (Jiang et al., 2004, 2005) and those which were also examined in HL-1 cells (R176Q/T2504M, R2474S and Q4201R (Jiang et al., 2005)). Single channel studies revealed that the mutants all displayed an increased sensitivity to luminal Ca^2+^, although the two N terminus mutations were 10-fold less sensitive than the others (Jiang et al., 2005). The mutants all had a lower threshold for spontaneous Ca^2+^ release (oscillations) when store load was varied with extracellular Ca^2+^ and the cells expressing the mutants all exhibited a reduced Ca^2+^ store content compared to WT (Jiang et al., 2005). The latter observation, whilst not invalidating the concept of increased sensitivity to luminal Ca^2+^ as part of the mechanism, does question the suitability of store ‘over’ load in the acronym for the hypothesis, even though as has been discussed above store overload can cause DADs (Diaz et al., 2004). There was no evidence of increased sensitivity to activating Ca^2+^ as ryanodine binding dose response curves to Ca^2+^ were essentially the same as WT. The increased response to luminal Ca^2+^ in the RyR2 mutants was not related to regulation by the CSQ complex since this is not expressed in HEK293 cells.

##### 4.2.3.4 Catecholaminergic polymorphic ventricular tachycardia-2 and regulation by luminal Ca^2+^

The CSQ mutations, which also cause the CPVT phenotype, have provided further insight into the proposed mechanism of regulation of RyR2 sensitivity to luminal Ca^2+^ via the CSQ/triadin/junctin complex. The CSQ mutant, with a D307H substitution, was characterised by adenoviral expression in rat myocytes and showed a reduced Ca^2+^ storage capacity and reduced spontaneous Ca^2+^ sparks (Viatchenko-Karpinski et al., 2004). Furthermore when paced and exposed to isoproterenol the myocytes displayed arrhythmic behaviour. Dialysis of the myocyte with citrate buffer, to reduce the SR store Ca^2+^ concentration, eliminated these extrasystolic transients (Viatchenko-Karpinski et al., 2004). A significant conformational change in the D307H protein structure was demonstrated, which may have contributed to both a reduced Ca^2+^ binding capacity and Ca^2+^-dependent binding to triadin and junctin (Houle et al., 2004). A knock-in mouse model of the D307H mutation caused CPVT. Myocytes from these animals were morphologically different from the WT including junctional SR of greater width and volume. Expression levels were also 2–6 fold higher than WT, although SR Ca^2+^ stores and NCX activity were unchanged. Whole cell patch clamp measurements showed changes in Ca^2+^ spark frequency, Ca^2+^ transients and Ca^2+^ current when challenged with isoproterenol and the membrane potential traces showed DADs (Dirksen et al., 2007).

Similar characterisation of an R33Q mutant showed that it had comparable Ca^2+^ binding capacity to the WT but when paced and exposed to isoproterenol myocytes displayed arrhythmic behaviour (Terentyev et al., 2006). Single channel studies revealed that R33Q lacked the ability to inhibit RyR2 regulation at low luminal Ca^2+^ concentrations suggesting that the mutation disrupted the regulatory association with RyR2 (via triadin and junctin) in some way (Terentyev et al., 2006). The underlying mechanism causing CPVT and RyR dysfunction in CSQ mutants could be altered Ca^2+^ buffering in the SR and susceptibility to luminal Ca^2+^ changes and/or dysregulation of RyR by the CSQ/junctin/triadin/complex (Bassani & Bassani, 2007; Gyorke & Terentyev, 2008). A junctin knock-out mouse had a high incidence of mortality (25% at three months despite structurally normal hearts) and when stimulated with isoproterenol demonstrated DADs (Yuan et al., 2007). Over expression of triadin in myocytes also caused arrhythmia (Terentyev et al., 2005; Kirchhof et al., 2007). These genes may be additional loci in which CPVT causing mutations might be found in the future.

##### 4.2.3.5 Cytoplasmic Ca^2+^ sensitivity

Caffeine sensitises RyR2 to activator Ca^2+^ and increases *P*_o_ in bilayers and in ryanodine binding assays (Meissner, 2002). MH mutations in RyR1 display enhanced caffeine sensitivity (Avila, 2005). RyR2, purified from an arrhythmogenic right ventricular cardiomyopathy (ARVC) patient heterozygous for G1885E/G1886 and G1885/G1886S, was compared for single channel characteristics to an ARVC patient with a RyR2 WT genotype, but whose heart was removed for transplant for cardiomyopathy (Milting et al., 2006). The mutant channels displayed sub-conductance states and at the lowest conductive state displayed a greatly enhanced *P*_o_ at pCa7.7, compared to WT (although RyR2 activity of this control may have been compromised by the cardiomyopathy (Milting et al., 2006). This suggested that increased sensitivity to cytosolic Ca^2+^ maybe the underlying dysfunction of RyR2 for this mutation. An increased sensitivity to Ca^2+^ and caffeine was demonstrated for S2246L, N4104K and R4497C mutations expressed in HEK293 cells or HL-1 cells, where it was also shown that there was no change in SR store Ca^2+^, which might have accounted for enhanced Ca^2+^ release (George et al., 2006). The characteristics of caffeine-dependent Ca^2+^ release varied greatly when N-terminus and central domain mutations were expressed in HEK293 cells (L433P, N2386I, R176Q, T2504M and R176Q/T2504M) (Thomas et al., 2004, 2005). N2386I and R176Q/T2504M showed greater sensitivity to caffeine at resting Ca^2+^. L433P, T2504 and R176Q/T2504M showed a profound loss of Ca^2+^ sensitive inhibition and SR store Ca^2+^ was unchanged in these experiments. However, others reported increased Ca^2+^ sparks and increased sensitivity to luminal Ca^2+^ for the L433P mutation (Jiang et al., 2005). Mouse R4496C (equivalent to human R4497C) was expressed in HEK293 cells and these showed a greater incidence of spontaneous Ca^2+^ release than cells expressing WT mRyR2. Increased basal channel activity was observed in both single channel studies and ryanodine binding experiments and the R4496C mutation showed a greater sensitivity to Ca^2+^ and caffeine (Jiang et al., 2002a).

#### 4.2.4 Consensus view of mutant characterisation

CPVT patients show a spectrum of phenotypic severity including a symptom-free cohort of carriers sharing the same genotype as symptomatic patients (Priori et al., 2002; Postma et al., 2005; Tester et al., 2005b). Possible reasons for this could be ‘rescue’ by co-expression of a polymorphism (Milting et al., 2006) or imprinting where a mutated gene from an affected adult is ‘silenced’ in an offspring and only the normal allele is expressed, as for RyR1 mutations (Zhou et al., 2006). It is proposed that the individual mutations may have different mechanisms by which they disrupt channel function (Thomas et al., 2005). Looking at the nature of the substituted amino acids suggests that there are different ways in which they may alter RyR function. Loss or insertion of a proline (P) a secondary structure helix breaker may disrupt protein folding. One such mutant — L433P, in separate but comparable studies using expression in HEK293 cells, showed altered caffeine-dependent activation in one (Thomas et al., 2004, 2005), but paradoxically increased Ca^2+^ spark activity and response to caffeine and increased sensitivity to luminal Ca^2+^ in another (Jiang et al., 2005) The reasons for this discrepancy are unclear at present. Nuclear magnetic resonance analysis of the peptide probe DP4 showed it to have two helical regions with a cluster of MH mutations (R2542W, I2453T, R2454C, R2458H or R2458C) located at the C-terminus of the first helix or in the loop connecting the two helices, suggesting a possible structural alteration in the relationship between the two helical regions (Bannister et al., 2006). Loss, addition or the swapping of charge on a residue could have implications for protein–protein interactions. In this respect it has been shown for the mouse R4496C mutation, expressed in HEK293 cells, that R4496E enhanced the characteristics shown by the mutant, whereas R4496K, which reversed the charge, showed the same characteristics as WT (Jiang et al., 2002a). The substitution or loss of serine (S) or threonine (T) residues have implications for phosphorylation and 3 such residues are lost and 12 created amongst the reported mutations (Thomas et al., 2006). It is suggested that the G1886S substitution creates a protein kinase C phosphorylation site (Milting et al., 2006). These considerations make it difficult to foresee a common molecular mechanism of dysfunction.

#### 4.2.5 Delayed after-depolarisations in catecholaminergic polymorphic ventricular tachycardia

The reports of normal function of hearts at rest suggest that there is no long-term change in the expression of the proteins that participate in maintaining the normal AP in CPVT. However, in ARVD2 patients there is some evidence of tissue remodelling of the right ventricle and one knock-in mouse model had some symptoms of cardiomyopathy (Kannankeril et al., 2006). Some patients with an abnormal ECG at rest have been reported (Tester et al., 2005b) and a slowed heart rate is also characteristic of CPVT (Choi et al., 2004; Postma et al., 2005), all of which suggests some modest compensation in some patients. However, there is no current experimental evidence to suppose that factors external to RyR2 function might contribute to DADs, as is the case in HF (see Section 4.1.4).

## 5 Therapy for heart failure and catecholaminergic polymorphic ventricular tachycardia

HF usually follows an increased demand (load), which cannot be met by the available healthy heart tissue. Without means of healing or replacing tissue damaged or lost, for example following a myocardial infarction (as might one day be possible with stem cell replacement) the best options currently available are to sustain the functional capacity of surviving tissue for as long as possible. The cells in this tissue, including the cardiomyocyte, are not passive bystanders. They bring to bear a veritable arsenal of genetic pathways intended for development, cell maintenance or short-term physiological flexibility but not ‘designed’ to mend a ‘broken’ heart (Swynghedauw, 2006). This striving to remodel the structural and functional components of the cell (and the whole organ) eventually leads to inappropriate compensation and ultimately ends in death either through pump failure or arrhythmia. Strategies for pharmaceutical intervention are to reduce the workload required of the heart and to improve the output.

Untreated, the mortality for CPVT is high (∼ 30% by the age of 30) with sudden death the first symptom in some instances (Priori et al., 2002). The genetics of CPVT have only recently been characterised and the familial pattern of inheritance and genetic screening has now enabled early diagnosis and specific treatment (Sumitomo et al., 2003; Napolitano et al., 2006). CPVT, which is essentially a disease of dysfunctional RyR2 activity, does not result in HF. It seems that arrhythmic episodes are not sufficient to destabilise the ECC fidelity in the long term even if they are not tolerated in the face of stress. The difference between HF and CPVT phenotypes suggests that RyR2 dysfunction is a likely consequence of the progression of HF, and not its cause. However, the link to DADs and fatal arrhythmia, as a result of RyR2 dysfunction in both conditions, merits the question as to whether strategies to improve RyR2 function may be of any benefit (Dulhunty et al., 2007; Santonastasi & Wehrens, 2007). In the treatment of HF, can targeting RyR2 do more than just prevent fatal arrhythmia at the end stage? Furthermore, do the dysfunctional mechanism(s) proposed for RyR2 in HF share any similarities to those for CPVT, which might offer insights into novel treatments for the latter?

Currently available therapeutic treatments for HF ameliorated symptoms and slow progression. The recommended treatment for chronic HF are angiotensin converting enzyme (ACE) inhibitor, β-blocker and diuretic often combined, dependent upon severity of symptoms (Weir & McMurray, 2005). β-blocker is the only pharmacological agent which can mollify the symptoms of CPVT and patients who do not respond are fitted with implantable defibrillators (Leenhardt et al., 1995; Priori et al., 2002). Prophylactic resynchronisation therapy is also indicated for some HF patients with symptoms of VT and fitting either a pacemaker or pacemaker-defibrillator in patients hospitalised for HF showed a reduced mortality in the following two years (Bristow et al., 2004; Feldman et al., 2005). A canine model of HF examined the effect of cardiac resynchronisation therapy. Treated hearts showed improvement in haemodynamic parameters. Isolated myocytes demonstrated reversal of characteristic markers of HF such as restitution of AP duration and resting membrane potential, as well as a recovery in the amplitude of Ca^2+^ transients (Nishijima et al., 2007).

### 5.1 Reducing the cardiac load in heart failure

#### 5.1.1 Left ventricular assist device

LVADs are used to support failing hearts awaiting donor transplantation. Their short-term use has been shown to reverse some of the changes in protein expression associated with end-stage HF and to aid recovery from the negative force frequency relationship by improving myocyte function (Dipla et al., 1998; Heerdt et al., 2000). In particular β-adrenergic responsiveness was restored (Dipla et al., 1998; Ogletree-Hughes et al., 2001). Significant increases in PP1 levels, reduced RyR hyperphosphorylation and increased FKBP12.6 bound (Marx et al., 2000), increased expression of LCC (Chen et al., 2002) and SERCA expression and function (Heerdt et al., 2000) have all been reported. The limited use of LVADs does suggest that alleviation of load on a failing heart can result in reverse remodelling and improve ECC. This gives hope that if a sufficiently effective therapeutic intervention were to be found to improve myocyte function even in end-stage HF, then some recovery of the heart may be achievable.

#### 5.1.2 Drug therapies for reducing cardiac load

ACE inhibitors, as a first line of therapy, do not act directly on cardiac tissue but have a vasodilatory action and work by reducing angiotensin II production and thereby reduce cardiac after-load and pre-load and encourage renal blood flow (Weir & McMurray, 2005). Diuretics, by decreasing the blood volume, can further aid this process. A group of compounds termed Ca^2+^-sensitizers also act to improve contractility, ideally without increasing oxygen demand and altering diastolic function (Brixius et al., 2005). One of these, levosimendan, has been approved for short-term treatment of decompensated HF and acts primarily to increase the sensitivity of troponin C to Ca^2+^ (Sorsa et al., 2001) but also activates ATP sensitive K^+^ channels and has a consequent vasodilatory action on the coronary circulation improving blood flow (Earl & Fitzpatrick, 2005).

### 5.2 β-blockers and ryanodine receptor type-2 function

β-blockers are a counter-intuitive therapy for HF since their *modus operandi* in the normal heart is to reduce contractility by preventing phosphorylation and consequent up-regulation of ECC (Lohse et al., 2003). Clinical trials have shown that β-blockers (bisprolol and carvedilol) are very effective in reducing arrhythmia and slowing the progression of HF (1994; McGavin & Keating, 2002; Poole-Wilson et al., 2003; Hori et al., 2004). In contrast, the use of β-agonists, such as dobutamine, to treat acute decompensation in HF, are only useful in the short term because they can be arrhythmogenic (Lohse et al., 2003).

In HF, the myocyte, because of the increased levels of circulating catecholamines, has already compensated by reducing β-receptor number (Bristow et al., 1982; Lohse et al., 2003; Tsukamoto et al., 2007). However these compensatory mechanisms result in an unco-ordinated state of phosphorylation as regards ECC proteins, with hypophosphorylation of PLB (Schwinger et al., 1999a; Dash et al., 2001; Schwinger & Frank, 2003) and hyperphosphorylation of RyR2 (Marks, 2003). The long term effect of β-blockade has broad spectrum consequences on ECC by increasing phosphorylation of PLB, hence increasing SERCA activity and elevating SR Ca^2+^ store concentrations (MacLennan & Kranias, 2003). RyR2 hyperphosphorylation is reported to be reduced and FKBP12.6 binding levels restored in a paced dog model treated with propranolol (Reiken et al., 2001; Doi et al., 2002) and in human heart muscle strips treated with carvedilol, metoprolol or atenolol (Reiken et al., 2003b). The AT1 receptor antagonist — valsartan, which inhibits noradrenaline release from the synaptic pool, also restored SR function by reducing by Ca^2+^ leak (Tokuhisa et al., 2006). There is evidence that patients prescribed the β-blocker carvedilol, had improved clinical outcomes in HF — the COMET study (Poole-Wilson et al., 2003) however, the underlying mechanism of advantage, based on comparison with metopropol, was uncertain when considered on the basis of relative efficacy of dose and selectivity for β-adrenergic subtype (Molenaar & Parsonage, 2005). Animal studies with carvedilol suggest its additional effectiveness might be ascribed to its antioxidant properties and stimulation of SERCA gene transcription (Koitabashi et al., 2005) as observed in rat cardiomyocytes. In a study of HF using a paced dog model, carvedilol was able to improve end-diastolic pressure four fold. RyR2 hyperphosphorylation was decreased and PP1, PP2 and FKBP12.6 association with the RyR2 macromolecular complex was restored. Single channel studies showed RyR2 channels with heterogeneous function from HF tissue with increased *P*_o_ but carvedilol reduced both *P*_o_ and heterogeneity (Xue et al., 2007). Results from a similar canine study showed that low dose carvedilol (insufficient to act through β-blockade) produced antioxidant effects that rescued RyR2 domain unzipping (Mochizuki et al., 2007).

Thus there is evidence that treatment with β-blockers improves RyR2 function along with other indicators of restitution of ECC. Their mechanism of action may be multifactorial. One outcome appears to restore the balance between phosphorylation/dephosphorylation that normally regulates ECC and this includes RyR2 function (Lohse et al., 2003). Another of the beneficial mechanisms of β-blockade may be a reduction in heart rate *per se.* A comparison of cilobradine (a sino atrial node inhibitor, which acts to slow heart rate) with metopropol, in a canine model of congestive HF, demonstrated improved haemodynamic parameters and restoration of SERCA and NCX expression to normal levels as a result of treatment (Cheng et al., 2007).

For the RyR2 CPVT mutants β-blockers (propranolol, metiletine, nadolol, atenolol, carteolol or metopropol (Leenhardt et al., 1995; Priori et al., 2001; Sumitomo et al., 2003; Aizawa et al., 2007) all improved mortality and reduced arrhythmic episodes, however, in one study, a high proportion of patients (37%) remained symptomatic (Priori et al., 2001) and in another 23% of patients died within 10 years (Sumitomo et al., 2003). 11 out of 13 CPVT2 patients with the CSQ mutation D307H (Lahat et al., 2001a) and 2 out of 3 with truncation mutations (Postma et al., 2005) were also effectively treated with propranolol. β-blockers are unfortunately not able to help a substantial number of patients in the long term.

### 5.3 Stabilising ryanodine receptor type-2 activity

#### 5.3.1 LCC agonists and ryanodine receptor type-2 function

Intravenous verapamil had a beneficial effect when given acutely to CPVT patients already taking β-blockers and increased the heart rate threshold at which exercise-induced arrhythmias appeared (Swan et al., 2005). Of interest were not the effects of verapamil on LCC activity, but the dose given was aimed at concentrations high enough to interact with RyR2 and inhibit ryanodine binding (Valdivia et al., 1990).

#### 5.3.2 JTV519 (K201)

Some animal models of HF have tested a compound first shown to ameliorate arrhythmia associated with DADs in paced dogs (Kaneko, 1994). This compound, known as JTV519 (K201), is an analogue of diltiazem — a LCC antagonist with a generalised spectrum of inhibition of several types of ion channel including K^+^ channels and RyR2 (Balasubramaniam et al., 2005). Diltiazem, but not nifedipine, suppressed caffeine and FK506 provoked arrhythmia in mouse hearts and myocytes demonstrating a link with spontaneous Ca^2+^ release (Balasubramaniam et al., 2005) and verapamil and diltiazem were able to decrease arrhythmic activity in a murine model of arrhythmia, induced by isoprenaline, (Balasubramaniam et al., 2004). The action of JTV519 suggested that it might also target RyR2 (Yano et al., 2003; Wehrens et al., 2004a), although like diltiazem it affects a number of ion channels, and action on LCC, Na^+^ and K^+^ channels have also been demonstrated (Kimura et al., 1999; Robinson et al., 2006; Loughrey et al., 2007). It is also reported to inhibit SERCA activity (Loughrey & Smith, 2006; Loughrey et al., 2007) and to act as an α_1_-adrenoreceptor blocker (Hasumi et al., 2007).

##### 5.3.2.1 The action of JTV519 on normal ryanodine receptor type-2 function

The effect of JTV519 is reported to involve the interaction of RyR2 with FKBP12.6 (Marks, 2002). In normal canine SR vesicles, JTV519 was shown to have no effect on SR Ca^2+^ uptake or on ryanodine binding (Kohno et al., 2003). Using canine SR vesicles the rate of Ca^2+^ leak was unmasked by the addition of thapsigargin to inhibit the Ca^2+^ uptake once the vesicle was full (Yano et al., 2003). JTV519 addition (1 µM) had no effect on the almost negligible release rate. The rate of release could be increased by the addition of FK506 to dissociate FKBP12.6 but when JTV519 was added with FK506 there was no such increase in leak rate and thus it was concluded that JTV519 could stabilise the channel in a similar way to FKBP12.6 (Yano et al., 2003). Ca^2+^ leak in canine SR vesicles, induced by peptide DPc10, was reversed by JTV519. However the amount of FKBP12.6 associated with SR vesicles was not changed by JTV519 so the inhibition of Ca^2+^ leak was not due to re-association of FKBP12.6 but a direct effect of JTV519 in stabilising the ‘zipper’ domain interaction (Oda et al., 2005). The binding site for JTV519 has been identified in a peptide of RyR2 corresponding to 2114–2149, which may interact with the central domain of the proposed zipper region (Yamamoto et al., 2007). When JTV519 was added to a dose response curve, for FK506 dissociation of FKBP12.6 from SR vesicles, 1 µM JTV519 shifted the curve marginally to the right but no change in the *k*_d_ was apparent (Yano et al., 2003). The action of JTV519 on SOICR was found to be independent of FKBP12.6 (Hunt et al., 2007). Spontaneous beats were found to be suppressed in both isolated myocytes, irrespective of the presence of FK506, and in HEK-293 cells expressing RyR2 alone or in combination with FKBP12.6. Decreased affinity for Ca^2+^ was also demonstrated in ryanodine binding experiments in response to JTV519 in the absence of FKBP12.6 (Hunt et al., 2007). Examination of the effect of JTV519 on ECC in rabbit ventricular cardiomyocytes demonstrated a reduction in ECC gain at 1 and 3 µM JTV519 (Loughrey et al., 2007). Increasing store Ca^2+^ resulted in diastolic spontaneous Ca^2+^ release events, which were reduced in frequency by JTV519 but the Ca^2+^ transient amplitude was unchanged. It was concluded that JTV519 could inhibit both RyR2 and SERCA and that at 3 µM JTV519 also reduced I_Ca_ through effects on LCC. There was no additional effect of JTV519 in myocytes over-expressing FKBP12.6 (Loughrey et al., 2007). These results mostly support the view that the action of JTV519 in reducing the activity of the RyR2 channel is a direct one and independent of FKBP12.6.

##### 5.3.2.2 JTV519 and heart failure

JTV519 has been shown to have beneficial effects in ischaemia and reconditioning. JTV519 improved energy metabolism (Kawabata et al., 2000) and the protective effects were attributed to inhibition of the post-ischaemic rise in Ca^2+^ (Hachida et al., 1999; Inagaki et al., 2000b). JTV519 has also been shown to activate PKCδ and up regulate its translocation to the plasma membrane — a part of the preconditioning response, which is protective in ischaemia, although the mechanism was not explored (Inagaki et al., 2000a). JTV519 acts on annexin V a cytoskeletal protein, which has Ca^2+^ binding and Ca^2+^ channel activity. Crystallisation of JTV519 with annexin V suggested that it bound to a hinge region (Kaneko et al., 1997a) and thereby inhibited Ca^2+^ movement (Kaneko et al., 1997b).

The action of JTV519 has been explored extensively in the context of the RyR2 hyperphosphorylation hypothesis. The original studies in a paced dog model showed that the prophylactic administration of JTV519 prevented the haemodynamic symptoms associated with HF. The isolated SR vesicles, from JTV519 treated animals, showed no abnormal Ca^2+^ leak, which was characteristic of HF in this model, and this was attributed to the lack of RyR2 hyperphosphorylation and the restitution of FKBP12.6 binding to levels seen in pre-paced hearts (Yano et al., 2003). It was uncertain whether the action of JTV519 was dependent upon rebinding of FKBP12.6 or if the rebinding occurred as a result of improved function of RyR2. This was specifically tested by one group using either FKBP12.6 null or +/− heterozygous knockout mice, which had structurally normal hearts (Wehrens et al., 2004a). The effect of JTV519 on RyR2 single channel function in +/− mice was to reduce *P*_o_. *P*_o_ of the −/− channel in the presence of JTV519 was greater than that of the untreated +/− mouse but regrettably this was not statistically compared to the untreated −/− control. Using programmed electrical stimulation to evoke arrhythmia in the same mice, or measuring spontaneous oscillations or isoprenaline-evoked Ca^2+^ waves in isolated myocytes, alternans, oscillation or waves could only be suppressed by JTV519 treatment in the +/− mice but not the −/− mice. Thus it was concluded that the presence of FKBP12.6 was necessary for the action of JTV519 (Lehnart et al., 2006) in this model.

The action of JTV519 on RyR2 zipper domain interaction has been explored in HF (Oda et al., 2005). Domain unzipping, measured using the fluorescent quencher method, and Ca^2+^ leak could be induced in canine SR vesicles using SIN-1, DPc10, cAMP (phosphorylation) or FK506 (Oda et al., 2005), both of which could be countered by JTV519 addition (Yano et al., 2005). In normal myocytes SIN-1 reduced cell shortening and decreased the peak Ca^2+^ transient and increased its duration. These effects were reversed by JTV519 (and the antioxidant edaravone — see Section 4.1.3) in a paced dog model (Yano et al., 2005).

JTV519 in animal models is clearly able to protect against the onset of HF but, although many studies have focussed on the changes associated with RyR2 function, it is not clear whether it acts specifically on RyR2 or through a broad spectrum of pharmacological activity against ion channels within the myocyte (Lehnart et al., 2005a). In addition to its action on the heart, JTV519 treatment had favourable effects in other tissues, which may contribute to the alleviation of symptoms in HF. It reversed hyperphosphorylation and promoted rebinding of FKBP12 to RyR1 in skeletal muscle, improving peripheral muscle function and relieving the fatigue associated with HF (Reiken et al., 2003a; Ward et al., 2003). JTV519 also enhanced renal haemodynamic and excretory properties by reducing haemodynamic load, an action ascribed to its effects on RyR2 expressed in kidney (Lisy & Burnett, 2006). Pulmonary veins have been implicated as a source of ectopic beats initiating atrial fibrillation. Experiments with isolated pulmonary vein myocytes showed JTV519 to be an anti-arrhythmic (Chen et al., 2008).

##### 5.3.2.3 JTV519 and catecholaminergic polymorphic ventricular tachycardia

JTV519 did not prevent arrhythmic episodes in the CPVT R4496C knock-in mouse (Liu et al., 2006). There has been one report that JTV519 can restore single channel function of CPVT mutant P2328S expressed in HEK293 cells. When phosphorylated by PKA in single channel studies the addition of JTV519 reduced *P*_o_ to that of WT, but unfortunately this study, which also included two C-terminus mutations, did not report any results for JTV519 on these (Lehnart et al., 2004). The P2328S mutation falls in the postulated zipper domain, and JTV519 has been shown to be effective in reversing the effects of DPc10 peptide-induced domain unzipping and in reversing the unzipped state of this domain in HF (Oda et al., 2005) and when the domain is unzipped by oxidation (Yano et al., 2005). Similar experiments using peptides to the N-terminus and C-terminus, spanning the respective R176Q or N4104K ARVD2 mutations, showed that JTV519 could reduce the incidence of DADs and reduce the frequency of Ca^2+^ sparks for the N terminus peptide but not for the C terminus (Tateishi et al., 2007). These experiments point at the possibility that JTV519 could be effective against CPVT mutations in the central zipper domain or its postulated interacting domain at the N-terminus and thereby benefit a select group of patients.

##### 5.3.2.4 Future therapies and ryanodine receptor type-2 stabilisation

Restored SR Ca^2+^ content is a marker of HF reversal in many models. Strategies to specifically target proteins concerned with increasing SR Ca^2+^ store content have all shown beneficial effects in ameliorating HF symptoms in animal models. These include over-expression of SERCA2a (Giordano et al., 1997; Baker et al., 1998; del Monte et al., 1999; Miyamoto et al., 2000; del Monte et al., 2001), ablation of PLB by antisense RNA (He et al., 1999; Eizema et al., 2000) and overexpression of phosphatase inhibitor P-1 to inhibit PP1 and enhance PLB phosphorylation (Pathak et al., 2005). Manipulating the expression of these proteins all represent potential targets for novel therapies. There is no reason why targeting RyR2 to stabilise the channel, both preventing diastolic Ca^2+^ release and increasing the SR Ca^2+^ store content should not be equally as effective (Dibb et al., 2007). It has been shown by using tetracaine, a RyR inhibitor, that reducing the *P*_o_ of RyR2 also reduces alternans (arrhythmic tendencies) in stimulated myocytes (Venetucci et al., 2006). In myocytes isolated from a canine model of HF, the observed reduction in Ca^2+^ store content could be reversed by the application of the RyR2 channel blocker ruthenium red (Belevych et al., 2007). JTV519 is currently in clinical trials for HF and it will be important to know if this fulfils the promise suggested by the animal studies. Analogues of JTV519, which maybe more RyR2-specific are in development (Lehnart et al., 2005a) and it will be interesting to observe if these are as effective in HF as their broad spectrum parent.

There is a range of severity in the phenotypic symptoms of CPVT and even within consanguineous families carrying the same mutation some probands can be symptom-free (Postma et al., 2005). The reasons for this are unclear. At the molecular level both CPVT1 and CPVT2 cause instabilities in RyR2 channel gating which lead to diastolic leak, which can be provoked experimentally by activation of RyR2 with pharmacological agents or exercise. There are clearly observable differences in the characteristics of individual mutations expressed in isolated cells, with possible different underlying mechanisms of RyR2 instability proposed (Thomas et al., 2006). So could an Ehrlich's ‘magic bullet’ exist for CPVT? — with ∼ 30% of patients intractable to β-blocker therapy one is certainly needed (Priori & Napolitano, 2005). A good example of such a bullet is the RyR1 inhibitory drug dantrolene. The RyR1 mutations show the same miscellany of substitution and clustered distribution as those for RyR2. By some fortuitous quirk, perhaps of molecular folding surrounding the binding site, and despite sharing an identical protein sequence for the proposed binding region (RyR1 L590-C609 and RyR2 L601-C619) (Palnitkar et al., 1999) dantrolene was thought to only affect RyR1 (Zhao et al., 2001). The binding site is outside of the N-terminus cluster of mutations (but able to stabilise zipper domain interactions) (Kobayashi et al., 2005). Importantly, its use to treat acute MH has reduced mortality to < 10%, which suggests that its action is generic and effective against most of the MH mutations (Krause et al., 2004; Rosenberg et al., 2007). It has now been reported that dantrolene cannot only bind to RyR2 but pretreatment can prevent ventricular tachycardia induced by epinephrine or exercise in mice with the R2474S knock-in CPVT mutation and prevent DADs in cardiomyocytes from these mice (Kobayashi et al., 2007). The relative potency of dantrolene for RyR1 versus RyR2 was not given.

Using existing therapies against the backdrop of knowledge of the mechanisms underlying RyR2 dysfunction allows for a strategic approach with potential new therapeutic compounds. JTV519 shows promise for HF but we do not know enough about its specificity of action on RyR2 nor have there been sufficient studies of its efficacy to know whether this promise extends to treatment of some CPVT mutations.

## 6 Ryanodine receptor type-2 arrhythmic mechanisms — reconciling structure, function and therapeutic targets

Overall ECC exhibits a tremendous capacity for compensatory regulation and is remarkably resilient both under normal physiological conditions and in the face of pathological assault and the same is true for RyR2. Although RyR2 has an inherent molecular defect in CPVT1, the finding of a similar phenotype in CPVT2 (CSQ mutation) suggests that RyR2 dysfunction is similarly compromised by dysregulation as also suggested in end-stage HF. The demise of RyR2 in both HF and CPVT have been considered jointly in this review, not least because RyR2 dysfunction, resulting in the diastolic leak and arrhythmia (DADs) in both conditions provide important clues to the underlying regulation and channel gating of RyR2 and its relationship to its microdomain environment. The understanding of these processes will provide the basis for therapeutic strategies.

Several mechanisms have been postulated to underlie dysfunction, perhaps they should be considered, not as mutually exclusive, but as if they form part of a continuum that underlies the regulation of RyR channel gating. It would not be difficult to construct a working hypothesis which relates molecular movement with channel (dys)function. When the channel is activated by Ca^2+^ this might result in a molecular reorganisation of the regulatory I-domain, which radiates to the pore to control channel gating (George et al., 2004). In turn, the movement of the I-domain may be regulated by modulator interactions with and within the zipper domain (Ikemoto & Yamamoto, 2002; Oda et al., 2005). These could be altered by phosphorylation, which promotes an allosteric change in the molecule affecting the affinity for FKBP12.6. Loosening the hold that FKBP12.6 has on RyR2 may be part of the normal flight or fight response (Jones et al., 2006; Wehrens et al., 2006). Perturb one of these molecular domain interactions by mutation and in the presence of phosphorylation the knock-on effect might be increased sensitivity to luminal or activator Ca^2+^, for example. As the pore opens (maybe like the opening of the iris of a camera (Serysheva et al., 1999)) perhaps sites sensitive to luminal Ca^2+^ are revealed (Jiang et al., 2005). As discussed, in Section 3.1, one of the many unknowns in RyR channel gating is how the four subunits of RyR2 work together and this may be a particularly important consideration in CPVT1 patients where the genotype is heterozygous and it might be expected that the resultant RyR2 tetramers will also be heterologous. How they function together is a disconcerting gap in our understanding of RyR structure/function and interpretation and interpolation of findings to the disease state.

Is RyR2 function and the inherent instability of CICR the ‘Achilles' heel’ of ECC? Indeed, in HF, because it is such a pivotal protein in ECC, acting as a regulator and coincidence detector of cellular events, RyR2 is buffeted by every type of stressful change encountered by the myocyte and in microcosm may merely reflect the myocyte's malaise. When faced with a chaotically regulated environment or wounded by a point mutation this magnificently robust protein may finally run out of possibilities to autoregulate coherently, when assaulted by phosphorylation. This might explain why broad spectrum strategies to rescue the myocyte such as β-blockade are successful in HF because they ‘treat’ ECC as a whole and partially successful in CPVT where phosphorylation maybe the trigger to arrhythmia.

Despite the intense spotlight on hyperphosphorylation and loss of FKBP12.6, this has not led to any real understanding of the relationship between RyR1/2 and FKBP12/12.6 or phosphorylation, in the context of the normal flight or fight response or the regulation of domain interactions in normal RyR2. In fact the molecular mechanism by which phosphorylation changes RyR2 function is uncertain and the experimental finding contradictory (see Section 2.4). The reasons why PP1 and PP2A are specifically lost from the RyR2 complex when they are present in the remainder of the cell in HF needs further investigation. Possibilities include changes in the association of their specific anchoring proteins for either the phosphatases or RyR2 or changes in their expression levels and understanding this mechanism might reveal a specific and novel therapeutic target for HF (Hulme et al., 2004). In CPVT any hesitancy in RyR2's molecular movement may be sufficient to desynchronise the normal open/closed molecular organisation and cause arrhythmia. Again there is little appreciation of the mechanism by which phosphorylation unmasks this instability. Preventing arrhythmia in CPVT may require a direct therapeutic regulation of RyR2 molecular activity — Ehrlich's ‘magic bullet’ (see Section 5.3.2.3).

The correction of RyR2 dysfunction in both point mutation carriers and HF patients and the development of novel therapeutic strategies is currently the focus of a much effort, particularly methods to prevent the onset of fatal arrhythmia. The efficacy of current and new therapeutic approaches is, in turn, providing valuable information on the underlying molecular mechanisms regulating RyR2 channel gating and function. In the final analysis, the pathological changes in RyR2 which cause spontaneous diastolic leak and arrhythmia must be understood from a perspective which correlates the dynamics of RyR2 channel gating with intramolecular movement, and this presents an intriguing challenge.

A third knock-in mouse model of CPVT has been reported for the central domain P2328S mutation. Comparison of WT, RyR^S/P^ and RyR^S/S^ genotypes demonstrated that gene dosage of the mutation increased the propensity to arrhythmia in myocytes and Langendorff heart preparations, particularly on the addition of isoproterenol. The physiological characteristics of CPVT were similar to the two models described in Section 4.2.2 describing C-terminal and N-terminal CPVT mutations (Goddard, C. A. et al. (2008). Physiological consequences of the P2328S mutation in the ryanodine receptor (*RyR2*) gene in genetically modified murine hearts. *Acta Physiol (Oxf)* 194, 123–140).
